# The Composition of Root-Associated Bacteria and Fungi of *Astragalus mongholicus* and Their Relationship With the Bioactive Ingredients

**DOI:** 10.3389/fmicb.2021.642730

**Published:** 2021-05-11

**Authors:** Yanmei Li, Yang Liu, Hui Zhang, Yan Yang, Gehong Wei, Zhefei Li

**Affiliations:** ^1^State Key Laboratory of Crop Stress Biology in Arid Areas, College of Life Science, Northwest A&F University, Xianyang, China; ^2^Shaanxi Key Laboratory of Agricultural and Environmental Microbiology, Northwest A&F University, Xianyang, China

**Keywords:** bioactive ingredients, rhizosphere, root endosphere, *Stenotrophomonas*, *Phyllobacterium*

## Abstract

*Astragalus membranaceus* (Fisch.) Bge. var. *mongholicus*, which is used in traditional Chinese medicine, contains several bioactive ingredients. The root-associated microbial communities play a crucial role in the production of secondary metabolites in plants. However, the correlation of root-associated bacteria and fungi with the bioactive ingredients production in *A*. *mongholicus* has not been elucidated. This study aimed to examine the changes in soil properties, root bioactive ingredients, and microbial communities in different cultivation years. The root-associated bacterial and fungal composition was analyzed using high-throughput sequencing. The correlation between root-associated bacteria and fungi, soil properties, and six major bioactive ingredients were examined using multivariate correlation analysis. Results showed that soil properties and bioactive ingredients were distinct across different cultivation years. The composition of the rhizosphere microbiome was different from that of the root endosphere microbiome. The bacterial community structure was affected by the cultivation year and exhibited a time-decay pattern. Soil properties affected the fungal community composition. It was found that 18 root-associated bacterial operational taxonomic units (OTUs) and four fungal OTUs were positively and negatively correlated with bioactive ingredient content, respectively. The abundance of *Stenotrophomonas* in the rhizosphere was positively correlated with astragaloside content. *Phyllobacterium* and *Inquilinus* in the endosphere were positively correlated with the calycosin content. In summary, this study provided a new opportunity and theoretical reference for improving the production and quality of in *A. mongholicus*, which thus increase the pharmacological value of *A. mongholicus.*

## Introduction

*Astragalus membranaceus* (Fisch.) Bge. var. *mongholicus* (Bge.) Hsiao (*A. mongholicus*) is a perennial herbaceous plant belonging to the legume family. The dried root of this plant, which is called Huangqi, is used in traditional Chinese medicine (Shao et al., 2004; Yan et al., 2016). More than 100 bioactive compounds, which are known as bioactive ingredients including flavonoids, saponins, polysaccharides, and amino acids, have been identified in Huangqi. In traditional Chinese medicine, Huangqi is used for treating tumors and diabetes (Fu et al., 2014). According to Chinese pharmacopeia criterion, the crude drug quality is determined based on the presence of one or more bioactive ingredients. Plant-associated microbes, including those inhabiting the plant tissue and rhizosphere, can improve plant health and regulate root metabolism (Philippot et al., 2013). Previous studies reported that the amount of bioactive ingredients varied widely depending on the cultivation location, seasons, and plant age. These factors determine the profile of microbes that have adapted to the host medicinal plant and also further promote plant-microbe interactions (Ma et al., 2002; Koberl et al., 2013). Various natural products are derived from microbes or microbial interactions with the host (Gunatilaka, 2006). The plant-associated microbiome, especially the complex microbial communities in the rhizosphere and root endomicrobiome, are directly or indirectly involved in the synthesis of bioactive components (Koberl et al., 2013). The plant microbiome may determine the efficacy of herbal medicines through the regulation of host metabolism (Huang et al., 2018).

The rhizosphere, an underground root-associated soil portion, is a special area surrounded and affected by plant roots and inhabited by numerous microorganisms (Dessaux et al., 2016). Plant roots secrete photosynthates into the environment known as root exudates, which can directly influence the rhizosphere composition (Bais et al., 2006). In turn, the rhizospheric microbiome exhibits various functions, including pathogen inhibition (Innerebner et al., 2011), nitrogen fixation, phosphorus solubilization, hormones production (Ali et al., 2009), and conferring plants with resistance to stress (Peiffer et al., 2013), to promote plant health or growth. Recent studies have reported that the reconstitution of the microbiome with microbes exhibiting beneficial properties in the rhizosphere and root could enhance plant health and survival rate (Berendsen et al., 2012; Meena et al., 2017). The microbial community structure is reported to be affected by abiotic and biotic factors, such as soil type (Bonito et al., 2014), geographical location (Edwards et al., 2015), plant species (Inceoglu et al., 2012), genotype (Philippot et al., 2013), and developmental stage (Xu et al., 2009; Chaparro et al., 2014). However, there is limited understanding of the impact of cultivation year on root-associated microbial communities, especially those associated with Chinese medicinal herbs.

The colonization of highly specific microbes in the medicinal plant promotes the production of unique and structurally divergent bioactive secondary metabolites (Qi et al., 2012). The secondary metabolism of medicinal plants is suggested to be correlated with their microbiome. *Pseudomonas*, *Bacillus*, and mycorrhiza fungi isolated from the rhizosphere and root endosphere promoted secondary metabolite production in the plants (Lugtenberg and Kamilova, 2009; Manoharachary et al., 2009). Zeng et al. (2013) demonstrated that the inoculation of arbuscular mycorrhizal fungi promotes plant growth and bioactive ingredient production in medicinal plants. *Bacillus subtilis* FZB24 promoted the production of picrocrocin, crocetin, and safranal in saffron (Sharaf-Eldin et al., 2008). The inoculation of rhizobacteria, such as *Azotobacter* and *Pseudomonas* enhances anethole production in *Pimpinella anisum* (Lugtenberg and Kamilova, 2009). Therefore, it is crucial to explore the effect of rhizosphere and endosphere microbial communities on the bioactive ingredient in Chinese medicinal herbs.

At present, *A*. *mongholicus* has successfully adapted to arid and semi-arid environments and is primarily cultivated on a large scale by farmers in the northern and northwestern regions of China (Jia et al., 2016). Previous study on *A*. *mongholicus* has focused on the medicinal value of bioactive ingredients for treating diseases (Huang et al., 2019). Meanwhile, some studies have focused on the rhizosphere microbial communities associated with different genotypes and successive cropping obstacles (Sun et al., 2017, 2018). However, the correlation of root-associated bacteria and fungi with the bioactive components of *A*. *mongholicus* in different cultivation years has not been completely elucidated. Soil properties markedly affect the composition of microbial communities. Therefore, this study explored the composition and structure of root-associated microbial communities and analyzed the effects of soil properties and cultivation years on root-associated bacteria and fungi to address several fundamental relevant questions: (1) How the microbial community structures vary in different cultivation years? (2) Whether soil properties or cultivation years has a greater impact on the microbial community; (3) Which microbes are related to the accumulation of bioactive ingredients in the root of *A. mongholicus*?

## Materials and Methods

### Study Area

The study area was located in the Zizhou County (37°31′7.8″ N, 110°03′57.66″ E), Shaanxi Province, China. The characteristics of the study area were as follows: altitude, 900–1,400 m; climatic conditions, temperate semi-arid continental climate; annual average temperature, 9.1°C; annual precipitation, 428.1 mm; average annual sunshine duration, 2,543 h. The soil in the study area was classified as loess (Calcaric Cambisol according to FAO classification). The major plant cultivated in the study area was *Astragalus mongholicus*. The soil was fertilized with organic fertilizer (3.75 t ha^–1^). The sampling site was subjected to similar fertilization and management practices.

### Experimental Design and Sampling Strategy

All the *A. mongholicus* samples were collected from the sampling site in August 2018. one-year-old (1-Y) plants denoted the plants cultivated in nursery substrates before transplantation. The 2-Y, 3-Y, 4-Y, and 5-Y plants refer to the plants transplanted and cultivated in the same zone in April 2016, 2015, 2014, and 2013, respectively. The sampling site comprised three plots where *A. mongholicus* was evenly cultivated as biological replicates. For each plot, three random soil samples from the topsoil (0–20 cm) in field without plants were collected using a drill. The samples were labeled as bulk soil. Three whole plant samples grown in different cultivation years were randomly uprooted using shovels. The remaining soil was manually removed by shaking the roots until approximately 1 mm soil was left attached to the roots. The samples were placed on ice in a cooler box and transported to the laboratory. Some soil samples were stored at −80°C until further analysis.

The soil layer with 1 mm thickness surrounding the root was defined as the rhizosphere soil (Beckers et al., 2017). To collect the rhizosphere soil directly from the root surface, the root was transferred to a sterile centrifuge tube containing sterile phosphate-buffered saline (PBS) solution (PBS-S; 130 mM NaCl, 7 mM Na_2_HPO_4_, 3 mM NaH_2_PO_4_ [pH 7.0], 0.02% Silwet L-77) (Xiao et al., 2017). The samples were vortexed for 15 s to release most of the rhizosphere soil. Next, the soil free from plant debris and large sediments was transferred into a fresh 50 mL centrifuge tube with 25 mL sterile PBS and centrifuged at 1,600 *g* and 4°C for 15 min. The supernatant was discarded and the sediments were centrifuged at 7,000 *g* for 10 min. This sample was labeled as rhizosphere soil and stored at −80°C.

The clean roots with the rhizosphere soil were placed in a fresh 50 mL centrifuge tube with 25 mL sterile PBS and sonicated for 30 s at 50–60 Hz using an ultrasonic cleaner (KS-250DE, Shanghai, China) at least five times. Next, the roots were washed with sterile distilled water for 5 min, surface-sterilized with 75% ethanol for 1 min and 1% NaClO for 30 s, and rinsed 10 times with sterile distilled water (Priyadharsini and Muthukumar, 2017; Kearl et al., 2019). To determine the efficacy of root surface sterilization, 10 μL of the final suspension was inoculated on Luria-Bertani agar medium. The sterile roots were stored at −80°C for DNA extraction of root entophytic microbiota. In total, 45 samples were obtained from bulk soil, rhizosphere soil, and root samples (3 repetitions × 5 cultivation years × 3 compartments).

### Analysis of Soil Characteristics

For soil properties analyses, the soil samples were collected by removing the plant materials, homogenized, air-dried and passed through a 2 mm sieve. The soil physicochemical properties, including soil water content (SWC), pH, organic matter (OM), total carbon (TC), total phosphorus (TP), total nitrogen (TN), total potassium (TK), available phosphorus (AP), available potassium (AK), ammonium nitrogen (NH_4_^+^-N), and nitrate nitrogen (NO_3_^–^-N) were analyzed according to the methods reported by Bao (2000).

### DNA Extraction, PCR Amplification, and Sequencing

DNA was extracted from 0.5 g soil or 0.05 g root using the Fast DNA^®^ SPIN kit for soil (MP Biomedicals, CA, United States) and the Power Plant^®^ DNA isolation kit (Mo Bio, CA, United States), respectively, following the manufacturer’s instructions. DNA concentration and purity were determined using a NanoDrop 2000 spectrophotometer (Thermo Scientific, Waltham, MA, United States) and 1% (*w/v*) agarose gel electrophoresis (Bio-Rad, CA, United States). The 16S rRNA gene amplicon library was generated using the PCR primers 799F (ACMGGATTAGATACCCKG) and 1193R (ACGTCATCCCCAC CTTCC), which amplify the V5–V7 region (Bulgarelli et al., 2012). The ITS1 amplicon library was generated using the primers 1737F (GGAAGTAAAAGTCGTA ACAAGG) and 2043R (GCTGCGTTCTTCATCGATGC) (Jiao et al., 2018). PCR was performed in a 50 μL reaction mixture comprising 0.5 μL of each primer (50 pmol), 25 μL of 2.5X Hot Master Mix (5-primer), 2 μL of DNA template, and 22 μL of sterile water. The PCR conditions were as follows: 94°C for 2.5 min (initial denaturation), followed by 30 cycles of 94°C for 30 s (denaturation), 55°C for 40 s (annealing), and 68°C for 40 s (extension), and a final extension step of 68°C for 7 min. The samples were stored at 4°C (Horton et al., 2014). The contamination in the PCR products was examined using gel electrophoresis with a 2% agarose gel at 120 V for 30 min. The PCR products were gel-purified using the QIAquick gel extraction kit (Qiagen, Dusseldorf, Germany). The sequencing libraries were generated using the Ion Plus fragment library kit (48 reactions, Thermo Scientific). The library quality was assessed using the Qubit@ 2.0 Fluorometer (Thermo Scientific). Finally, the library was sequenced on an IonS5XL^TM^ platform (Thermofisher Inc., Massachusetts, United States) and 400 bp single-end reads were generated by Novogene (Beijing, China).

### Sequence Processing

The raw data were quality-filtered according to the method reported by Caporaso et al. (2010). The chimeric sequences were removed using USEARCH with the Uchime tool (Edgar et al., 2011) based on the reference databases Silva^1^ and Unite^2^. The sequence abundance data matching “Chloroplast” and “Mitochondria” were removed from the data sets (Beckers et al., 2017). For sequence analysis, the operational taxonomic units (OTUs) clustered at 97% similarity (Edgar, 2010) were assigned to each sample with a 12-bp barcode using a script derived from the QIIME^3^ pipeline (Caporaso et al., 2010). The RDP classifier with an 80% confidence threshold was used to assign the taxonomic groups for the representative sequences of each OTU (Edwards et al., 2015). The OTU abundance dataset was normalized using a standard sequence number corresponding to the sample with the least sequences. All subsequent analyses were performed based on the normalized data.

### Determination of Astragalosides and Flavonoids

The astragaloside and flavonoid contents were determined according to the protocol reported by Xu et al. (2007) with minor modifications. Briefly, 0.5 g dried powder (sieved through a 0.23 mm mesh) was placed in a 15 mL centrifuge tube with 10 mL methyl alcohol (chromatographic grade). The samples were sonicated for 120 min at 40 Hz and 100 W (KS-250DE ultrasonic cleaner). The supernatant was passed through a 0.22 μm hydrophobic membrane loaded into a 2.0 mL sample bottle using a sterile syringe after centrifuging twice at 12,000 rpm for 15 min. The standard samples (5.0 mg each) of astragalosides (astragalosides I, astragalosides II, astragalosides III, and astragalosides IV) or flavonoids (formononetin, calycosin-7-*β*-glucoside, calycosin, and ononin) were dissolved in methanol (final volume: 10 mL) to a concentration of 500 μg mL^–1^. Methanol was used to dissolve 2.0 mg of mixed standard samples to prepare 5, 10, 25, 50, 100, and 200 μg mL^–1^ solutions, which were used for preparing the final concentration gradient.

The liquid chromatography-tandem mass spectrometry (LC-MS) analysis of the samples was performed following the protocols of Yu et al. (2007) with modifications. The samples were loaded into an LC-MS system (API 2000, AB Sciex, MA, United States) equipped with a chromatographic guard column Wondasil^®^ C18 (4.6 mm × 150 mm, 5 μm), a triple quadrupole mass spectrometer detector (QQQ), and an electrospray ion source. MS was performed in the positive ion (H^+^/Na^+^) mode to separate the parent and sub ions according to the relative molecular mass of each standard. The liquid phase elution gradient, sample quality spectrum parameters, and mass spectrum operating parameters were similar to those used in previous studies (Jiao et al., 2015; Tuan et al., 2015).

### Statistical Analysis

The normal distribution of the data was determined using the Shapiro-Wilk method. Homoscedasticity of variances was analyzed using Bartlett test analysis (Beckers et al., 2017). The analysis was based on the normalized dataset. To assess the microbial diversity and abundance, the alpha diversity indices (Chao1 and richness) were calculated using QIIME^4^ pipeline, while the beta diversity was estimated by the Bray-Curtis distance between different sample groups. The Bray-Curtis distance index is applied to analyze the difference in abundance observed between the same taxa across pairs of samples. All statistical analyses were performed using the R V3.6.0 environment^5^. The results were visualized using the “ggplot2” package (Wickham, 2009). The alpha diversity indices and soil properties of different groups were analyzed using analysis of variance (ANOVA). The correlation between the variables was examined based on Spearman’s correlation coefficient and visualized using the “corrplot” package (Wei et al., 2013). The package “ggpubr” was used for the linear regression analysis.

Non-metric multidimensional scaling (NMDS) based on Bray-Curtis distance was implemented using the “vegan” package (Dixon, 2003). This package was also used for the analysis of similarities (Anosim) and permutational multivariate analysis of variance (Adonis) (Anderson, 2001), which were performed with 999 permutations (Oksanen et al., 2020). The redundancy analysis (RDA) with 999 permutations was performed using the “vegan” package to analyze the effects of soil properties and cultivation years on microbial composition. Differentially abundant OTUs were analyzed using the “EdgeR” package with the generalized linear models to investigate the enriched and depleted OTUs in different compartments. OTUs with significant variation over the five cultivation years were identified using the R package “MaSigPro” (Conesa et al., 2006). The shared taxa were selected from overlapping OTUs in Venn diagrams using the “Venndiagram” package (Chen and Boutros, 2011).

## Results

### Soil Chemical Properties and Bioactive Ingredients in Different Cultivation Years

The soil properties varied in different cultivation years (Table 1). Soil primary properties (SWC, AK, AP, TN, TP, TK, OM, and NO_3_^–^-N) significantly decreased with the cultivation year. In contrast, soil pH significantly increased with the cultivation year. The TC content initially decreased but later increased from the forth year. In contrast, the NH_4_^+^-N content initially increased but later decreased from the forth year.

**TABLE 1:** Soil properties analysis in different cultivation years.

**soil properties**	**SWC**	**pH**	**AK**	**AP**	**TN**	**TC**	**TP**	**TK**	**OM**	**NO_3_^–^-N**	**NH4^+^-N**
1-Y	0.13 ± 0.01 a	8.24 ± 0.01 c	183.30 ± 2.52a	161.28 ± 0.82a	0.75 ± 0.05a	18.65 ± 0.24 a	1.09 ± 0.02a	10.12 ± 0.11a	10.49 ± 0.11a	69.23 ± 1.38a	1.38 ± 0.06ab
2-Y	0.08 ± 0.01 d	8.81 ± 0.06 b	60.90 ± 2.22c	11.78 ± 1.82b	0.34 ± 0.07b	15.70 ± 0.09c	0.65 ± 0.01b	9.37 ± 0.21b	5.95 ± 0.81b	1.52 ± 0.38b	0.92 ± 0.37b
3-Y	0.09 ± 0.01 c	9.03 ± 0.02 a	99.70 ± 26.66b	12.14 ± 1.183b	0.31 ± 0.02b	15.04 ± 0.82c	0.60 ± 0.02c	9.32 ± 0.14b	5.74 ± 0.63b	0.86 ± 0.31b	2.36 ± 1.09a
4-Y	0.09 ± 0.06 c	9.01 ± 0.02 a	60.57 ± 1.73b	11.38 ± 2.19b	0.27 ± 0.04b	15.63 ± 0.18c	0.59 ± 0.02c	9.67 ± 0.18b	4.67 ± 0.06c	0.51 ± 0.11b	2.43 ± 0.46a
5-Y	0.12 ± 0.01b	9.01 ± 0.03 a	48.93 ± 0.67b	10.55 ± 0.33b	0.30 ± 0.04b	17.13 ± 0.03b	0.67 ± 0.01b	9.71 ± 0.35ab	5.49 ± 0.53bc	0.41 ± 0.17b	0.33 ± 0.11b
*F*	41.26	385.1	84.08	8701	69.54	54.49	757.7	8.72	77.97	8524	10.68
*P*	<0.001	<0.001	<0.001	<0.001	<0.001	<0.001	<0.001	<0.001	<0.001	<0.001	<0.001

*The ANOVA analysis and Turkey were used to compare the soil properties across different cultivation years. SWC, Soil water content; AK, Available potassium; AP, available phosphorus; TN, Total nitrogen; TC, Total carbon; TP, Total phosphorus; TK, Total potassium; OM, Organic matter. NO_3_^–^-N, Nitrate nitrogen; NH_4_^+^-N, Ammonium nitrogen.*

**FIGURE 1:**
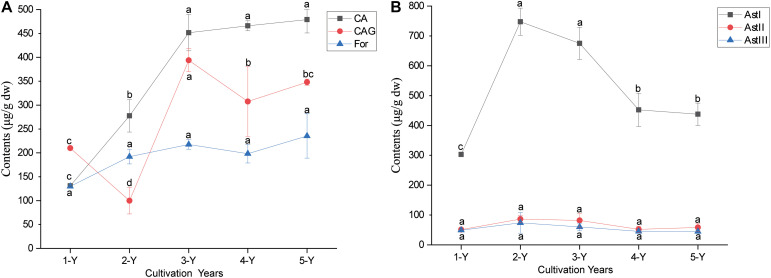
Changes in the root bioactive ingredients depending on the cultivation years. The contents of flavonoids **(A)** and astragalosides **(B)** in different cultivation years. Different letters in each error bar represent significant differences (ANOVA; *P* < *0.05*; *n* = 3). For, formononetin; CAG, calycosin-7-β- glucoside; CA, calycosin; AstI, astragalosides I; AstII, astragalosides II; AstIII, astragalosides III; ANOVA, analysis of variance.

The ANOVA of bioactive ingredient contents revealed that the cultivation year significantly affected the contents of astragaloside I (AstI), calycosin (CA), and calycosin-7-*β*-glucoside (CAG) (*P* < 0.05) (Figure 1). The AstI content peaked in the second year and decreased thereafter, whereas the CAG content was the lowest in the second year and increased thereafter. The CA content in the *A. mongholicus* root increased with every cultivation year. Additionally, there was no significant difference the contents of astragaloside II (AstII), astragaloside III (AstIII), and formononetin (For) in the different cultivation years (*P* < 0.05) (Figure 1).

### Microbial Community Composition and Alpha Diversity

**FIGURE 2:**
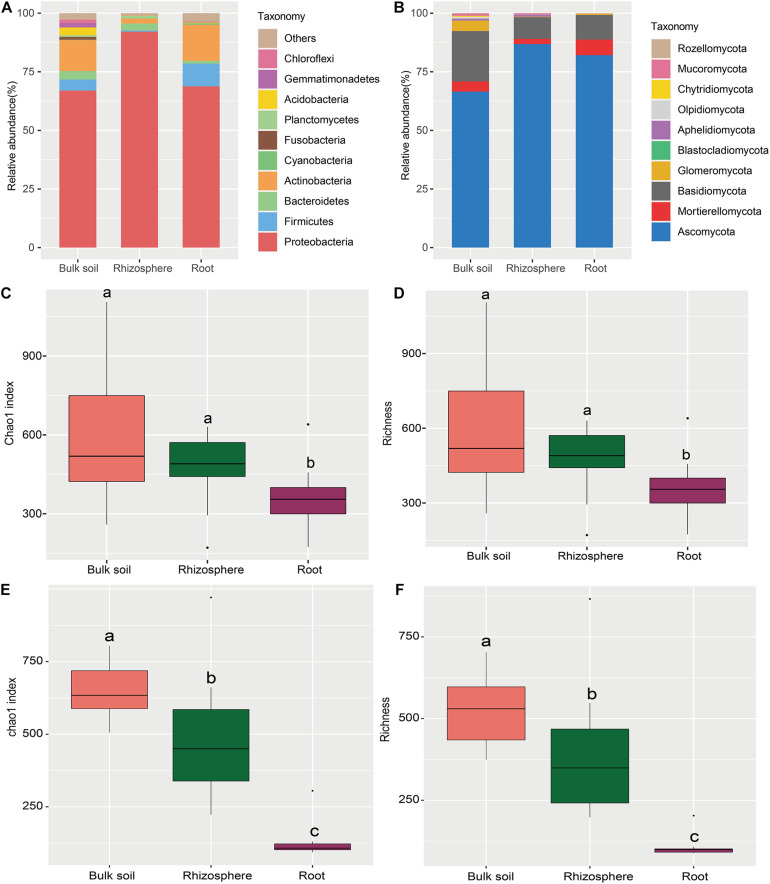
Taxonomic profile and alpha diversity of bacterial and fungal communities in the sampling compartments of *Astragalus mongholicus*. **(A)** The relative abundance of top 10 bacterial **(A)** and fungal **(B)** phyla. Boxplots of the alpha diversity indices (Chao1 index and richness) of root-associated bacteria **(C,E)** and fungi **(D,F)** in the three compartments. Different letters indicate significant differences (*P* < *0.05*; Kruskal-Wallis test).

The sequencing of 16S rRNA and ITS amplicons yielded 3,430,765 bacterial and 3,605,770 fungal quality-filtered reads. In total, 2,716,911 bacterial reads (79.19% sequences) were classified into different bacterial phyla (median, 80,113; range, 54,321–80,302 sequences per sample). Meanwhile, 2,626,765 fungal reads, which accounted for 72.84% of sequences, were classified into fungal phyla (median, 80,117; range, 80,007–80,250 sequences per sample). After homogenization, 3599 bacterial and 2,985 fungal OTUs were identified across all samples. Proteobacteria, Firmicutes, and Actinobacteria were the major bacterial phyla in the bulk soil and roots, whereas Proteobacteria and Bacteroidetes were the predominant phyla in the rhizosphere (Figure 2A). The relative abundance of Ascomycota, Mortierellomycota, and Basidiomycota, which were the predominant fungal phyla in three sampling compartments, accounted for almost 92.37–99.25% of total reads (Figure 2B), while Glomeromycota and Mortierellomycota dominated in the bulk soil and root endosphere, respectively (Figure 2B).

The relative abundance of different microbial taxa varied in the different cultivation years (Supplementary Figure 1). For bacteria, the abundance of Proteobacteria, increased from 1-Y to 4-Y cultivation year in bulk soil, while the abundance of Bacteroidetes increased from 3-Y to 5-Y in the rhizosphere (Supplementary Figure 1A). For fungi, Ascomycota phylum enriched in the 3-Y in bulk soil. However, the relative abundance of Basidiomycota and Glomeromycota increased in the 4-Y and 2-Y, respectively. Additionally, Ascomycota in the rhizosphere showed the increasing trend from 1-Y to 4-Y (Supplementary Figure 1B). Worthily, Mortierellomycota was enriched in the root endosphere of 5-Y cultivation year.

The alpha diversity indices (Chao1 and richness) of bacteria and fungi were comparatively analyzed in the three compartments by Wilcoxon rank sum test (Figure 2). Pairwise comparision of Chao1 and richness significantly varied in the three sampling compartments, except for those of bacterial community in the bulk soil and rhizosphere (Figures 2C–F and Supplementary Tables 1, 2), and the Chao1 and richness in the three sample compartments decreased from bulk soil to root (Figures 2C–F). Although no significant change of alpha diversity was observed in different cultivation year through ANOVA analysis (Supplementary Figures 2, 3), the bacterial alpha diversity in rhizosphere and bulk soil showed decreased trend from 1-Y to 4-Y (Supplementary Figure 2). The same trend of fungal alpha diversity was found in rhizosphere from 1-Y to 3-Y (Supplementary Figure 3). However, the alpha diversity of bacterial and fungal communities in root endosphere remained almost constant in different cultivation years (Supplementary Figures 2, 3).

### Microbial Community Variation in Different Sampling Compartments and Cultivation Years

**FIGURE 3:**
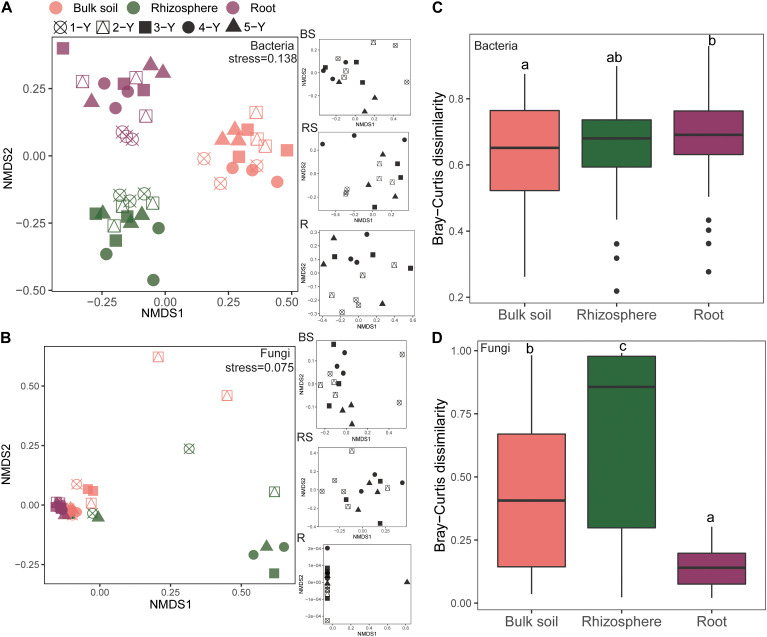
Beta diversity of bacterial and fungal communities in different sampling compartments. Non-metric multidimensional scaling (NMDS) plots of root-associated bacterial **(A)** and fungal **(C)** community composition across different sites based on the Bray-Curtis distance. The right insets represent the three soil–root compartments of bacteria and fungi community. Boxplots of root-associated bacterial **(B)** and fungal **(D)** community similarity among the three compartments. Different letters indicate significant differences (*P* < *0.05*; Kruskal-Wallis test). BS, bulk soil; RS, rhizosphere; R, root endosphere.

The NMDS plot was visualized to analyze the microbial community composition difference based on the Bray-Curtis distance in different compartments and cultivation years (Figure 3). The bacterial community in different compartments formed distinct clusters (Figure 3A). Consistently, the Adonis and Anosim results (based on *n* = 999) revealed that the bacterial community composition significantly varied among the sample compartments (Adonis: *R*^2^ = 0.366, *P* = *0.001*; Anosim: *R* = 0.930, *P* = *0.001*; Table 2). The comparative analysis of Bray-Curtis dissimilarity of bacterial communities revealed that the similarity of bacterial community in the three sampling compartments can be ranked in the following order: bulk soil < rhizosphere soil < root (Figure 3C). With respect to the fungal community, Adonis and Anosim analysis based on the Bray-Curtis distance measures suggested that compartments affected the fungal community composition (Adonis, *R*^2^ = 0.198, *P* = 0.001; Anosim, *R* = 0.245, *P* = 0.001; Table 2). The fungal community similarity in the rhizosphere was significantly higher than that in the bulk soil and root endosphere (Figure 3D).

**TABLE 2:** Adonis and Anosim statistical analyses of the microbial community composition of all samples based on Bray-Curtis distance.

	**Bacteria**				**Fungi**			

**Factors**	**Adonis**		**Anosim**		**Adonis**		**Anosim**	
	**R^2^**	***P***	**R**	***P***	**R^2^**	***P***	**R**	***P***
Compartments	0.366	0.001***	0.930	0.001***	0.198	0.001***	0.245	0.001***
Years	0.098	0.001***	–0.026	0.696	0.607	0.589	–0.029	0.935
Compartments*Years	0.172	0.001***			0.159	0.414		
	**Years**							
Bulk soil	0.370	0.046*	0.201	0.036*	0.387	0.054	0.148	0.049*
Rhizosphere	0.531	0.001***	0.625	0.001***	0.219	0.706	–0.116	0.760
Root	0.377	0.029*	0.228	0.061	0.279	0.510	0.052	0.256

*Compartments include bulk soil, rhizosphere and root endosphere. Years analysis was controlled the same compartment.*

Adonis, permutational multivariate analysis of variance using distance matrices; Anosim, analysis of similarities. Significance analysis was based on 999 permutation tests. *P < *0.05*; **P < *0.01*; ***P < *0.001*.

The cultivation year contributed 9.80% (*P* = 0.001) of the variations for bacterial community in Bray-Curtis distance measures (Table 2). Then the statistical analysis of bacterial and fungal community composition in different cultivation years in three sampling compartments based on the Bray-Curtis distance indicated that the cultivation year had significant effects (*P* < *0.05*) on bacterial community composition in rhizopshere (Adonis: *R*^2^ = 0.531; Anosim: *R* = 0.625) and bulk soil (Adonis: *R*^2^ = 0.370; Anosim: *R* = 0.201) (Table 2). And then the comparative analysis of Bray-Curtis dissimilarity of bacterial communities among different cultivation years demonstrated that the similarity of bacterial community was significantly lower in the first year than other cultivation years (Supplementary Figure 4A). However, the cultivation year did not affect (*P* > *0.05*) the fungal community composition (Table 2). And the fungal community similarity was not significantly different among the cultivation years (Supplementary Figure 4B).

### Time-Decay Pattern of Root-Associated Bacteria and Fungi

**FIGURE 4:**
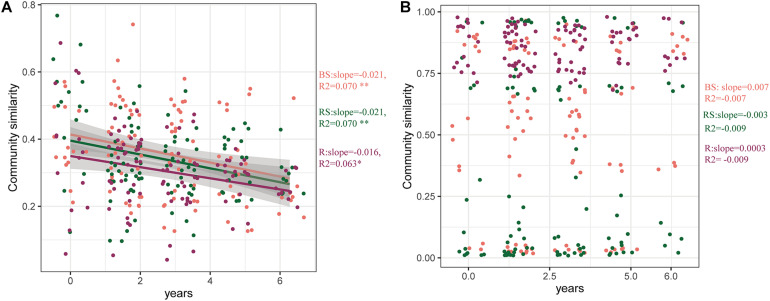
Time-decay pattern of the bacteria, fungi, and whole taxa. Time-decay patterns of bacterial **(A)** and fungal **(B)** taxa in the three compartments. The rate of community turnover across time (slopes). The correlation coefficient (R^2^) is provided. Significance of the linear fitting model is represented with ^∗∗^*P* < *0.01* and ^∗^*P* < *0.05*. BS, bulk soil; RS, rhizosphere; R, root endosphere.

To further explore the effects of cultivation year on the microbial communities, the time-decay patterns of bacterial and fungal communities were estimated (Figure 4). Consistent with previous results, the bacterial communities in the three sampling compartments exhibited a significant time-decay pattern (*P* < 0.05) (Figure 4A). The bacterial communities in the bulk soil and rhizosphere exhibited a stronger rate of decay (slopes = −0.021) than those in the root (slopes = −0.016). However, the fungal community did not exhibit a significant time-decay pattern. This is because the cultivation year did not affect the fungal microbiome (Figure 4B).

### Effect of Environmental Factors on Microbiome Structures of *A. mongholicus*

**FIGURE 5:**
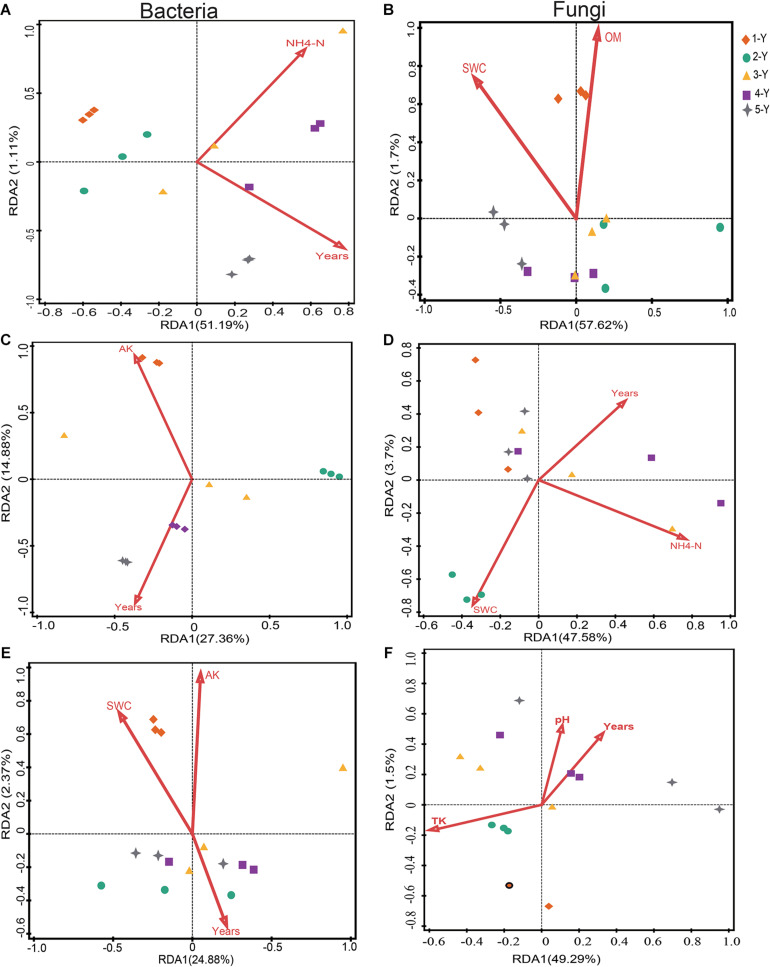
Redundancy analysis (RDA) of the correlation between soil properties and the composition of the bacterial and fungal communities. The effects of soil properties on bacterial **(A**,**C,E)** and fungal **(B**,**D,F)** communities in the bulk soil, rhizosphere, and root.

Redundancy analysis analysis was performed to investigate the potential correlation between the top 10 phyla of microbiomes and environmental factors (including cultivation year) (Figure 5 and Supplementary Tables 3, 4). The bacterial community of the bulk soil was significantly influenced by the cultivation year (*P* = 0.011) and NH_4_^+^-N concentration (*P* = 0.022), which accounted for 53% of the total variance (*P* = 0.009) (Figure 5A and Supplementary Table 3). Similarly, the cultivation year influenced the bacterial community in the rhizosphere (*P* = 0.01), which accounted for 25.7% of the variation (Figure 5C and Supplementary Table 3). This indicated that the cultivation year was the predominant influencing factor for the bacterial community structure in bulk soil and rhizosphere. Compared with the bulk soil and rhizosphere bacterial communities, the root endosphere bacterial communities were relatively stable. Soil properties and cultivation year did not significantly affect the root endosphere bacterial communities. However, the cultivation year contributed to 16.3% of the variation in the microbial composition of the root endosphere (Figure 5E and Supplementary Table 3).

The impact of environmental factors on the fungal community composition was analyzed by employing RDA. Soil OM and SWC were the main edaphic factors, which accounted for 59.32% of the variation in the fungal community composition of the bulk soil (Figure 5B and Supplementary Table 4). SWC (*P* = 0.019) and NH_4_^+^-N (*P* = 0.042) significantly affected the rhizospheric fungal community composition (Figure 5D and Supplementary Table 4). RDA revealed that TK (variation = 21.8%, *P* = 0.047) was the major environmental factor influencing the root fungal community composition (Figure 5F and Supplementary Table 4). These data suggested that cultivation year had stronger effect on bacterial community in bulk soil and rhizosphere, and that the bacterial microbiomes in root endosphere were stable and minimally influenced by the cultivation year. However, soil properties were the dominant factors influencing the fungal community composition.

### Correlation Between Root-Associated Bacteria and Fungi and Bioactive Ingredients

In total, 176 and 69 bacterial OTUs were enriched in the rhizosphere and root, respectively (Supplementary Figure 5). These bacterial OTUs enriched in the rhizosphere belonged to eight phyla encompassing 66 genera (Supplementary Figure 5A). OTUs enriched in the roots belonged to five phyla and encompassed 38 genera (Supplementary Figure 5B). Moreover, 121 and 43 fungal OTUs were significantly enriched in the rhizosphere and root, respectively (Supplementary Figure 6). The most of significantly differential fungal OTUs were in the rhizosphere belonged to Ascomycota and Unidentified_fungi (Supplementary Figure 6A). Interestingly, almost all fungal OTUs that were significantly enriched in the root were Unidentified_fungi (Supplementary Figure 6B).

The analysis using the “MaSigPro” package found that 182 bacterial and 32 fungal OTUs with significant variation among different cultivation years were detected in the rhizosphere and root (Supplementary Figures 7, 8). Of these, 92 significant rhizospheric bacterial OTUs were divided into the following three clusters: cluster I, 45 OTUs that were not equally distributed among different cultivation years; cluster II, 44 OTUs that were enriched in the first year; cluster III, three OTUs that were significantly enriched in the third year (Supplementary Figure 7A). In the root endosphere, 90 OTUs were classified into the following two clusters (Supplementary Figure 7B): cluster I, 74 OTUs that were significantly upregulated in the first year; cluster II, 16 OTUs that were unevenly distributed in the last 4 years. The abundance of variable fungal OTUs in the rhizosphere across different cultivation years was the highest in the first year (Supplementary Figure 8A). The significant variable fungal OTUs in the root were divided into the following two clusters: cluster I, 7 OTUs that were unevenly enriched in different cultivation years; cluster II, 6 OTUs that were upregulated in the third and fourth year (Supplementary Figure 8B).

**FIGURE 6:**
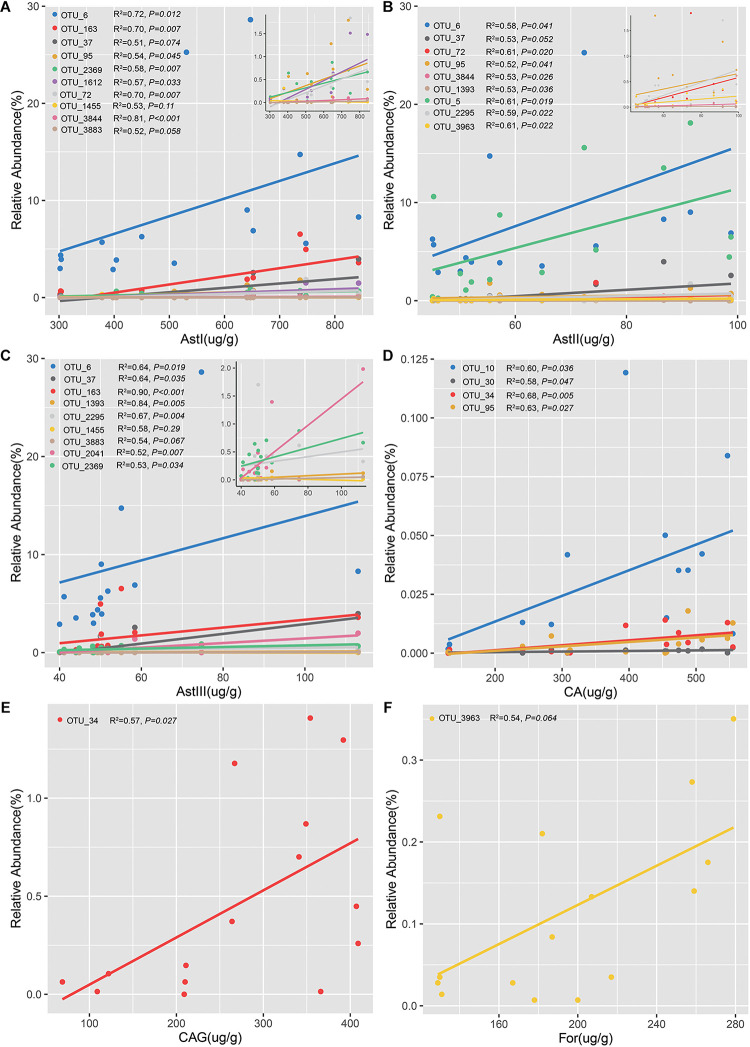
Linear regression analysis of the relative abundance of operational taxonomic units (OTUs) significantly and positively correlated with the contents of bioactive ingredients. Analysis of OTUs with astragalosides I **(A)**, astragalosides II **(B)**, astragalosides III **(C)**, calycosin **(D)**, calycosin-7-glucoside **(E)**, and formononetin **(F)**. Figures in the upper right of each panel show the OTUs with a low relative abundance related to the bioactive ingredients.

The OTUs confirmed by both differential species and the ones that varied significantly across different cultivation years in rhizsophere and root were defined as the shared OTUs (Supplementary Figure 9). Of the 57 rhizospheric bacterial OTUs, 15 were positively correlated with the content of bioactive ingredients (Figure 6 and Supplementary Tables 5–7). Linear regression analysis revealed that 10 OTUs were positively correlated with AstI content (Figure 6A and Supplementary Table 5). In particular, the abundance of *Sphingopyxis* (OTU_3844) was significantly correlated with AstI content (*R*^2^ = 0.81, *P* < *0.001*). Additionally, the AstII content was correlated with the relative abundance of 9 OTUs (Figure 6B and Supplementary Table 5). The relative abundance of 9 OTUs was positively correlated with AstIII content (Figure 6C and Supplementary Table 5). *Lysobacter* (OTU_163) was positively correlated with AstIII content (*R*^2^ = 0.90, *P* < *0.001*). Besides, OTU_6 (*Stenotrophomonas*) was positively correlated with the contents of astragalosides (AstI, AstII, and AstIII). Spearman’s correlation analysis indicated that only 3 (3/15) bacterial OTUs of root endosphere promoted bioactive ingredient production (Figures 6D–F and Supplementary Table 6). Additionally, the abundance of OTU_34 was positively correlated with CA (*R*^2^ = 0.68, *P* = *0.0053*; Figure 6D) and CAG contents (*R*^2^ = 0.57, *P* = *0.027*; Figure 6E). Furthermore, the relative abundance of *Xanthomonadaceae* (OTU_3963) was positively correlated with For content (Figure 6F). The fungal OTUs were negatively correlated with CA and AstII contents (Supplementary Table 7). These findings suggested that the bacterial community, especially the rhizobacterial community, played a more important role than fungal microbiomes in the accumulation of bioactive ingredients.

## Discussion

### Differential Responses of Bacterial and Fungal Communities of *A*. *mongholicus* to Soil Properties and Cultivation Years

Soil properties have been considered to be one of the key factors affecting the microbial composition (Philippot et al., 2013; Reinhold-Hurek et al., 2015). In our study, the soil properties significantly varied among different cultivation years. But RDA results suggested that cultivation year was the pivotal factor influencing bacterial community structures, rather than soil properties. The phylum-level of root-associated bacterial and fungal community composition displayed different trends across the cultivation years. The observed abundance of Proteobacteria, Actinobacteria, and Bacteroidetes, which were the most abundant phyla in the rhizosphere, varied across cultivation years. This is consistent with the results of previous studies (Bulgarelli et al., 2012; Lundberg et al., 2012). Compared with the rhizospheric composition, Actinobacteria enriched in bulk soil and Proteobacteria depleted in the bulk soil across different cultivation years. Consistently, the bacterial community composition exhibited a time-decay pattern in the three sampling compartments (*P* < 0.05). Previous studies have demonstrated that the composition of rhizospheric bacterial and fungal communities of many plants (such as *Arabidopsis*, sugarcane, pea, wheat, sugar-beet, and soybean) is dependent on the plant developmental stage (Baudoin et al., 2002; Houlden et al., 2008; Micallef et al., 2009; Chaparro et al., 2014). Plant developmental stages are classified based on the following criteria: seedling stage, development stage, flowering stage, and seed setting stage. It has been reported that root-associated microbiome composition was shaped by plant growth stage because of the changes in root exudates (Berg, 2009; Stringlis et al., 2018). Therefore, cultivation year is a critical factor shaping the composition and structure of bacterial communities.

Compared with that of bulk soil and rhizosphere, the bacterial community composition of the root endosphere was relatively stable in *A. mongholicus*. The cultivation year and soil properties did not affect the root endophyte microbiome. The stability of the plant root endosphere microbiome across cultivation years can be attributed to the strong selection and filtering effects of the roots. Rhizodeposition and root exudates from the host plant affect the microbial composition. Thus, specific bacteria are required for the ingress and establishment in the root owing to a root selective barrier (Bais et al., 2006; Lugtenberg and Kamilova, 2009; Reinhold-Hurek et al., 2015; Beckers et al., 2017). Because of that, Spearman correlation analysis of shared OTUs and bioactive ingredients revealed that the degree of correlation between the abundance of bacterial genera and bioactive metabolites in the rhizosphere was higher than that in the root.

Although the fungal phyla Ascomycota and Basidiomycota dominated in the rhizosphere and root and fluctuated across the cultivation years, the fungal composition was significantly affected by the soil properties. Previous studies have demonstrated that soil nutrient availability determine the soil fungal composition (Lauber et al., 2008; Hu et al., 2014; Tedersoo et al., 2014). RDA revealed that the composition of the fungal microbiomes in bulk soil, rhizosphere, and root endosphere were significantly coordinated by soil OM, SWC, and TK. Voriskova and Baldrian (2013) reported that microbes, such as Ascomycota are involved in the degradation of soil OM. In this study, soil OM decreased with the cultivation year, which can be attributed to the increased abundance of Ascomycota phylum in the rhizosphere and root with time. Conversely, OM is correlated with the structure and functions of the microbial community (Manoharan et al., 2017). Previous studies have reported that soil OM was the major factor determining the bacterial and fungal community composition in the Alpine landscape (Zinger et al., 2011) and global topsoil (Bahram et al., 2018). The results of this study indicated that SWC varied depending on the cultivation year. The fungal community structure exhibits plasticity as moisture fluctuation which induces a rapid turnover of fungal populations (Kaisermann et al., 2015). SWC can directly or indirectly influence the microbial community composition by regulating oxygen concentrations and nutrient availability (Drenovsky et al., 2004). The enhanced levels of soil moisture decrease the gas diffusion rates, which directly affect the microbial physiology and activities. Additionally, the enhanced levels of soil moisture increase the liquid diffusion rates and consequently provide microorganisms with substrates (Blagodatsky and Smith, 2012). TK was the major factor determining the fungal community structure. This finding is consistent with that of a previous study, which reported that TK content determined the fungal community structure (Yao et al., 2017).

### Correlation Between Shared OTUs and the Contents of Bioactive Ingredients

Recent studies have demonstrated that various bioactive ingredients are derived from microbes or through microbial interaction with the host (Gunatilaka, 2006; Koberl et al., 2013). In this study, rhizobacteria were associated with astragaloside content, while flavonoid accumulation correlated with the microbiota in the root endosphere. Astragalosides are reported to directly shape the specific microbial community structure within and around the root (Huang et al., 2019). Spatial heterogeneity of flavonoid exudation is observed along the root. Additionally, the soil microbes alter the flavonoid contents (Hassan and Mathesius, 2012). Previous studies have demonstrated that several microbes associated with medicinal plants promote plant-microbe interactions. These microbes mediate several functions, including the growth, physiology, and secondary metabolite production in the host (Koberl et al., 2013; Huang et al., 2018). Therefore, we inferred that astragaloside and flavonoid exudation within or around the roots may selectively recruit specific microbes that could produce substances to regulate plant growth and promote bioactive ingredient accumulation.

Among the shared taxa in the rhizosphere, OTU_6 (which was identified as *Stenotrophomonas*) was significantly correlated with the content of AstI, AstII, and AstIII. *Stenotrophomonas* colonized the rhizosphere of many plant species, including oilseed rape, maize, potato, cabbage, mustard, beet, solanum, eggplant, and chili (Berg et al., 1996; Jankiewicz et al., 2012; Naz, 2012; Audipudi, 2015; Patel and Saraf, 2017). Several studies have reported that *Stenotrophomonas* can promote plant germination and growth and suppress plant pathogens by stimulating the production of indole acetic acid (phytohormone), chitinase, and siderophore (Berg and Martinez, 2015; An and Berg, 2018; Zhang et al., 2020). For example, *S. maltophilia* AVP27 improves the growth of chili plants by regulating the production of plant hormones (Audipudi, 2015; Mukherjee and Roy, 2016). The increased abundance of *Stenotrophomonas* can contribute to the growth of *Polygonum cuspidatum* growth through a combining effect of emodin (Zhang et al., 2020). In this study, *Stenotrophomonas* was detected in the *A*. *mongholicus* rhizosphere. Hence, we speculated that *Stenotrophomonas* was recruitmented in the rhizosphere may facilitate *A*. *mongholicus* growth and stimulate metabolite secretion through various pathways. The secreted metabolites can support *Stenotrophomonas* growth in the rhizosphere and improve astragaloside production.

Our results further showed that AstI and AstII were positively correlated with the relative abundance of *Sphingopyxis* in the rhizosphere. *Sphingopyxis* has been isolated from various habitats, including natural soil, underground water, sediments of contaminated rivers, oil-contaminated soil, seawater, landfill soil and ginseng field (Ushiba et al., 2003; Lee et al., 2008; Verma et al., 2015; Chaudhary and Kim, 2016; Chaudhary et al., 2017a,b). Therefore, we speculated that *Sphingopyxis* was widespread and had a variety of potential functions. Additionally, microorganisms associated with flavonoid accumulation in this study mainly belonged to the genera *Phyllobacterium* and *Inquilinus*. *Inquilinus*, which was reported to be isolated from the ginseng field soil, belongs to the class Alphaproteobacteria (Jung et al., 2011). Other ecological effects and functions of Inquilinusit remained still unknown and needed to be further investigated. *Phyllobacterium*, which is reported to increase the plant growth, fruit yield, or mineral content in strawberry, *Capsicum annuum*, and cucumber, is considered a plant probiotic (Ipek et al., 2014; Silva et al., 2014; Pii et al., 2016; Rueda et al., 2016). Moreover, *Phyllobacterium* can promote the production of some bioactive compounds in strawberries (Flores-Felix et al., 2015, 2018). Therefore, *Phyllobacterium* could be a potentially beneficial endophyte strain for enhancing *A. mongholicus* growth and quality. Other special rhizobacteria and endosphere bacteria also increase the bioactive ingredient contents. Future studies must verify the function of specific microbial strains and elucidate the mechanisms underlying specific strains and bioactive ingredient accumulation in *A. mongholicus*.

## Conclusion

This study investigated the structure of the rhizosphere and root endosphere microbiomes of the medicinal plant *A. mongholicus* across different cultivation years and elucidated the correlation between the microbial communities, bioactive ingredient and soil properties. Apart from the bacterial microbiome in the root endosphere remaining relatively stable, the community composition of bacteria and fungi varied across the cultivation years. The cultivation years had a higher impact on the bacterial community composition than soil properties which strongly affected the fungal community composition. Meanwhile, the abundance of *Stenotrophomonas* in the rhizosphere was positively correlated with the content of astragaloside, while that of *Phyllobacterium* and *Inquilinus* was positively correlated with the CA content in the roots. Furthermore, the content of bioactive ingredients was positively correlated with the abundance of some bacteria in the rhizosphere and root endosphere. This study revealed the complex association among root-associated bacteria and fungi, bioactive ingredients, and soil properties, providing us a new opportunity to improve the production and quality of *A. mongholicus*.

## Data Availability Statement

The datasets presented in this study have been uploaded into the NCBI Sequence Read Archive (SRA) with the accession number (Bacteria: PRJNA686217 and Fungi: PRJNA687561).

## Author Contributions

YLi collected the samples, performed the experiments, analyzed the data, and prepared the manuscript. YLiu, YY, and HZ collected the samples and conducted the experiments. GW and ZL conceived the study and revised the manuscript. All authors reviewed the results and approved the final version of the manuscript.

## Conflict of Interest

The authors declare that the research was conducted in the absence of any commercial or financial relationships that could be construed as a potential conflict of interest.

## Footnotes

1 https://www.arb-silva.de

2 https://unite.ut.ee

3 http://qiime.org/scripts/index.html

4 http://qiime.org/index.html

5 http://www.r-project.org
