# Supplementary material for: The Composition of Root-Associated Bacteria and Fungi of Astragalus mongholicus and Their Relationship With the Bioactive Ingredients
Source: Front Microbiol. 2021 May 11;12:642730. doi: 10.3389/fmicb.2021.642730 (PMC8147693; doi:10.3389/fmicb.2021.642730)
Supplement: Supplementary file 1 [file Data_Sheet_1.docx]

**Supplementary materials**

**Table S1** Pairwise comparisons of Chao1 index and Richness between different compartments in bacteria community using Wilcoxon rank sum test.

| Compartment 1 | Compartment 2 | Mean of compartment 1 | Mean of compartment 2 | *P* value | sig. |
| --- | --- | --- | --- | --- | --- |
| Chao1 index | | | | | |
| Bulk soil | Rhizosphere | 626.135 | 594.706 | 0.32 | - |
| Bulk soil | Root | 626.135 | 444.566 | <0.01 | ** |
| Rhizosphere | Root | 594.706 | 444.566 | <0.01 | ** |
| Richness | | | | | |
| Bulk soil | Rhizosphere | 519 | 490 | 0.36 | - |
| Bulk soil | Root | 519 | 355 | <0.01 | ** |
| Rhizosphere | Root | 490 | 355 | <0.01 | ** |

‘-’ represented no significant difference.*, *P < 0.05*; **, *P < 0.01*; ***, *P < 0.001*.

**Table S2** Pairwise comparisons of Chao1 index and Richness between different compartments in fungi community using Wilcoxon rank sum test.

| Compartment 1 | Compartment 2 | Mean of compartment 1 | Mean of compartment 2 | P value | sig. |
| --- | --- | --- | --- | --- | --- |
| Chao1 index | | | | | |
| Bulk soil | Rhizosphere | 633.815 | 449.75 | <0.001 | *** |
| Bulk soil | Root | 633.815 | 107.688 | <0.001 | *** |
| Rhizosphere | Root | 449.75 | 107.688 | <0.001 | *** |
| Richness | | | | | |
| Bulk soil | Rhizosphere | 530 | 349 | <0.001 | *** |
| Bulk soil | Root | 530 | 99 | <0.001 | *** |
| Rhizosphere | Root | 349 | 99 | <0.001 | *** |

*, *P < 0.05*; **, *P < 0.01*; ***, *P < 0.001*.

**Table S3** The results of environmental factors interpretation for bacterial community after redundancy analysis (RDA) with 999 permutations.

| Envfit | Explains (%) | Contribution (%) | F | P |
| --- | --- | --- | --- | --- |
| Bacteria-Bulk soil | | | | |
| Years | 31.4 | 36.1 | 5.9 | 0.011 |
| NH_4_^+^-N | 20.9 | 24.1 | 5.3 | 0.022 |
| total |  |  | 6.6 | 0.009 |
| Bacteria-Rhizosphere | | | | |
| AK | 16.6 | 16.6 | 2.6 | 0.095 |
| Years | 25.7 | 25.7 | 5.3 | 0.01 |
| total |  |  | 4.4 | 0.009 |
| Bacteria-Root | | | | |
| SWC | 6.6 | 7.4 | 0.9 | 0.442 |
| AK | 4.6 | 5.0 | 0.6 | 0.564 |
| Years | 16.3 | 18.0 | 2.5 | 0.125 |
| total |  |  | 1.4 | 0.234 |

**Table S4** The results of environmental factors interpretation for fungal community after redundancy analysis (RDA) with 999 permutations.

| Envfit | Explains (%) | Contribution (%) | F | P |
| --- | --- | --- | --- | --- |
| Fungi-Bulk soil | | | | |
| SWC | 26.2 | 26.6 | 4.6 | 0.041 |
| OM | 31.6 | 32.1 | 9.0 | 0.006 |
| total |  |  | 8.2 | 0.008 |
| Fungi-Rhizosphere | | | | |
| NH_4_^+^-N | 28.1 | 28.3 | 5.1 | 0.042 |
| SWC | 26.2 | 26.4 | 9.7 | 0.019 |
| Years | 12.5 | 12.6 | 2.5 | 0.135 |
| total |  |  | 2.3 | 0.117 |
| Fungi-Root | | | | |
| TK | 21.8 | 22.1 | 4.7 | 0.047 |
| Years | 16.2 | 16.5 | 2.5 | 0.09 |
| pH | 11.4 | 11.5 | 1.9 | 0.199 |
| total |  |  | 3.6 | 0.056 |

**Table S5** Spearman correlation analysis of the shared bacterial OTUs in rhizosphere with the contents of bioactive ingredients.

| Bacteria_Rhizopshere | | Correlation analysis | | | | | | Taxonomy | | | | |  |
| --- | --- | --- | --- | --- | --- | --- | --- | --- | --- | --- | --- | --- | --- |
| OUT_ID | RA | AstI | AstII | AstIII | CA | CAG | For | Phylum | Class | Order | Family | Genus |  |
| **OTU_5** | 28.20% | - | **0.62** | - | - | - | - | *Proteobacteria* | *Gammaproteobacteria* | *Xanthomonadales* | *Xanthomonadaceae* | |  |
| **OTU_6** | 38.98% | **0.72** | **0.58** | **0.64** | - | - | - | *Proteobacteria* | *Gammaproteobacteria* | *Xanthomonadales* | *Xanthomonadaceae* | *Stenotrophomonas* |  |
| OTU_8 | 2.56% | - | - | - | - | - | - | *Proteobacteria* | *Gammaproteobacteria* | *Pseudomonadales* | *Pseudomonadaceae* | *Pseudomonas* |  |
| OTU_9 | 13.98% | - | - | - | - | - | - | *Proteobacteria* | *Gammaproteobacteria* | *Pseudomonadales* | *Pseudomonadaceae* | *Pseudomonas* |  |
| OTU_15 | 4.85% | - | - | - | - | - | - | *Proteobacteria* | *Alphaproteobacteria* | *Rhizobiales* | *Rhizobiaceae* | *Unidentified_Rhizobiaceae* |  |
| OTU_18 | 23.25% | - | - | - | - | - | - | *Proteobacteria* | *Gammaproteobacteria* | *unidentified_Gammaproteobacteria* | *Burkholderiaceae* |  |  |
| OTU_25 | 3.27% | -0.59 | -0.62 | - | - | - | - | *Proteobacteria* | *Alphaproteobacteria* | *Rhizobiales* | *Rhizobiaceae* | *Ochrobactrum* |  |
| **OTU_37** | 2.80% | **0.51** | **0.53** | **0.64** | - | - | - | *Planctomycetes* | *Phycisphaerae* | *Phycisphaerales* | *Phycisphaeraceae* |  |  |
| OTU_41 | 3.28% | - | - | - | - | - | - | *Bacteroidetes* | *Bacteroidia* | *Flavobacteriales* | *Flavobacteriaceae* | *Flavobacterium* |  |
| OTU_47 | 3.79% | - | - | - | -0.53 | - | - | *Proteobacteria* | *Gammaproteobacteria* | *Xanthomonadales* | *Rhodanobacteraceae* | *Pseudofulvimonas* |  |
| **OTU_72** | 1.08% | **0.70** | **0.61** | - | - | - | - | *Proteobacteria* | *Alphaproteobacteria* | *Sphingomonadales* | *Sphingomonadaceae* | *Sphingopyxis* |  |
| **OTU_95** | 1.84% | **0.54** | **0.52** | - | **0.63** | - | - | *Proteobacteria* | *Gammaproteobacteria* |  |  |  |  |
| OTU_124 | 1.39% | - | - | - | - | - | - | *Proteobacteria* | *Alphaproteobacteria* | *Caulobacterales* | *Caulobacteraceae* | *Phenylobacterium* |  |
| **OTU_163** | 6.71% | **0.70** | **-** | **0.90** | - | - | - | *Proteobacteria* | *Gammaproteobacteria* | *Xanthomonadales* | *Xanthomonadaceae* | *Lysobacter* |  |
| OTU_193 | 0.23% | - | **-** | - | - | - | - | *Proteobacteria* | *Alphaproteobacteria* |  |  |  |  |
| OTU_218 | 0.09% | -0.67 | -0.61 | - | - | - | - | *Proteobacteria* | *Alphaproteobacteria* | *Rhizobiales* | *Hyphomicrobiaceae* | *Hyphomicrobium* |  |
| OTU_237 | 0.30% | - | **-** | - | - | - | - | *Proteobacteria* | *Alphaproteobacteria* |  |  |  |  |
| OTU_239 | 0.49% | - | **-** | - | - | - | - | *Proteobacteria* | *Alphaproteobacteria* | *Sphingomonadales* | *Sphingomonadaceae* | *Novosphingobium* |  |
| OTU_498 | 0.08% | - | **-** | - | -0.73 | - | -0.77 | *Proteobacteria* | *Alphaproteobacteria* |  |  |  |  |
| OTU_602 | 1.83% | - | **-** | - | -0.72 | -0.57 |  | *Proteobacteria* | *Alphaproteobacteria* | *Rhizobiales* | *Rhizobiaceae* | *Ensifer* |  |
| OTU_710 | 0.12% | -0.64 | **-** | - | -0.61 | - | -0.62 | *Proteobacteria* | *Gammaproteobacteria* |  |  |  |  |
| OTU_807 | 0.08% | -0.65 | **-** | - | - | - | -0.52 | *Gemmatimonadetes* | *unidentified_*  *Gemmatimonadetes* | *Gemmatimonadales* | *Gemmatimonadaceae* | *unidentified_*  *Gemmatimonadaceae* |  |
| OTU_1009 | 2.51% | - | **-** | - | - | - | -0.53 | *Proteobacteria* | *Alphaproteobacteria* | *Rhizobiales* | *Rhizobiaceae* | *Mesorhizobium* |  |
| **OTU_1393** | 0.16% | - | **0.53** | **0.84** | - | - | - | *UnidentifiedBacteria* | *Bacteria* |  | *Unidentified_Bacteria* | *Unidentified_bacteria* |  |
| **OTU_1455** | 0.03% | **0.53** | **-** | **0.58** | - | - | - | *Planctomycetes* | *Phycisphaerae* | *Tepidisphaerales* |  |  |  |
| **OTU_1612** | 1.40% | **0.57** | **-** | **-** | - | - | - | *Proteobacteria* | *Gammaproteobacteria* | *Xanthomonadales* | *Xanthomonadaceae* | |  |
| **OTU_2041** | 1.61% | - | **-** | **0.52** | - | - | - | *Proteobacteria* | *Gammaproteobacteria* | *unidentified_Gammaproteobacteria* | *Burkholderiaceae* |  |  |
| OTU_2084 | 0.81% | - | **-** | **-** | - | - |  | *Proteobacteria* | *Gammaproteobacteria* | *Pseudomonadales* | *Pseudomonadaceae* | *Pseudomonas* |  |
| OTU_2223 | 3.06% | - | **-** | **-** | - | - | -0.59 | *Proteobacteria* | *Alphaproteobacteria* | *Caulobacterales* | *Caulobacteraceae* | *Phenylobacterium* |  |
| **OTU_2295** | 1.36% | - | **0.59** | **0.67** | - | - | - | *Proteobacteria* | *Gammaproteobacteria* | *unidentified_Gammaproteobacteria* | *Burkholderiaceae* | *Massilia* |  |
| **OTU_2369** | 1.63% | **0.58** | **-** | **0.53** | - | - | - | *Proteobacteria* | *Gammaproteobacteria* | *unidentified_Gammaproteobacteria* | *Burkholderiaceae* | *Alcaligenes* |  |
| OTU_2904 | 27.03% | -0.55 | **-** | - | - | - | - | *Proteobacteria* | *Gammaproteobacteria* | *Pseudomonadales* | *Pseudomonadaceae* | *Pseudomonas* |  |
| OTU_3026 | 0.24% | - | **-** | - | -0.54 | - | -0.53 | *Proteobacteria* | *Alphaproteobacteria* | *Sneathiellales* | *Sneathiellaceae* | *Ferrovibrio* |  |
| OTU_3109 | 0.49% | - | **-** | - | - | - | - | *Bacteroidetes* | *Bacteroidia* | *Sphingobacteriales* | *Sphingobacteriaceae* | *Sphingobacterium* |  |
| OTU_3179 | 0.20% | - | **-** | - | - | - | - | *Proteobacteria* | *Gammaproteobacteria* | *unidentified_Gammaproteobacteria* | *Burkholderiaceae* | *Verticia* |  |
| OTU_3189 | 2.49% | - | **-** | - | - | - | - | *Proteobacteria* | *Alphaproteobacteria* | *Caulobacterales* | *Caulobacteraceae* | *Brevundimonas* |  |
| OTU_3211 | 2.98% | - | **-** | - | - | - | - | *Proteobacteria* | *Gammaproteobacteria* | *unidentified_Gammaproteobacteria* | *Burkholderiaceae* |  |  |
| OTU_3525 | 0.87% | - | **-** | - | -0.56 | - | - | *Proteobacteria* | *Alphaproteobacteria* | *Sphingomonadales* | *Sphingomonadaceae* | *Altererythrobacter* |  |
| OTU_3729 | 1.08% | - | **-** | - | -0.66 | -0.68 | -0.57 | *Proteobacteria* | *Alphaproteobacteria* | *Caulobacterales* | *Caulobacteraceae* | *Phenylobacterium* |  |
| **OTU_3844** | 0.16% | **0.77** | **0.53** | - | - | - | - | *Proteobacteria* | *Alphaproteobacteria* | *Sphingomonadales* | *Sphingomonadaceae* | *Sphingopyxis* | |
| OTU_3871 | 0.43% | -0.82 | -0.52 | - | -- | - | - | *Proteobacteria* | *Gammaproteobacteria* | *Pseudomonadales* | *Pseudomonadaceae* | | |
| **OTU_3883** | 0.05% | **0.52** | **-** | **0.54** | **-** | **-** | **-** | *Proteobacteria* | *Gammaproteobacteria* | *unidentified_Gammaproteobacteria* | *Burkholderiaceae* |  | |
| **OTU_3963** | 0.51% | - | **0.61** | **-** | **-** | **-** | **0.54** | *Proteobacteria* | *Gammaproteobacteria* | *Xanthomonadales* | *Xanthomonadaceae* | | |
| OTU_4044 | 1.15% | - | **-** | **-** | **-** | **-** | **-** | *Proteobacteria* | *Gammaproteobacteria* | *unidentified_Gammaproteobacteria* | *Burkholderiaceae* | *Massilia* | |
| OTU_4132 | 0.08% | - | - | - | - | -0.59 | - | *Proteobacteria* | *Alphaproteobacteria* | *unidentified_Gammaproteobacteria* | *Acetobacteraceae* | *Roseomonas* | |
| OTU_4136 | 1.29% | - | - | - | - | -0.61 | -0.71 | *Proteobacteria* | *Alphaproteobacteria* | *Rhizobiales* | *Rhizobiaceae* | *Mesorhizobium* | |
| OTU_4182 | 0.05% | - | - | - | - | - | -0.54 | *Proteobacteria* | *Gammaproteobacteria* | *unidentified_Gammaproteobacteria* | *Burkholderiaceae* |  | |
| OTU_4257 | 0.39% | - | - | - | - | - | - | *Proteobacteria* | *Gammaproteobacteria* | *unidentified_Gammaproteobacteria* | *Burkholderiaceae* |  | |
| OTU_4262 | 0.45% | - | - | - | -0.64 | -0.61 | -0.59 | *Proteobacteria* | *Alphaproteobacteria* | *Caulobacterales* | *Caulobacteraceae* | *Phenylobacterium* | |
| OTU_4315 | 0.03% | - | - | - | -0.78 | -0.85 | - | *Proteobacteria* | *Gammaproteobacteria* | *unidentified_Gammaproteobacteria* | *Procabacteriaceae* | *Procabacter* | |
| OTU_4374 | 0.20% | - | - | - | - | - | - | *Proteobacteria* | *Alphaproteobacteria* | *Sphingomonadales* | *Sphingomonadaceae* | *Altererythrobacter* | |
| OTU_4516 | 0.67% | - | - | - | - | - | - | *Proteobacteria* | *Alphaproteobacteria* | *Rhizobiales* | *Beijerinckiaceae* | *Bosea* | |
| OTU_4616 | 0.60% | -0.53 | - | - | -0.56 | - | - | *Proteobacteria* | *Alphaproteobacteria* | *Rhizobiales* | *Rhizobiaceae* | *Ochrobactrum* | |
| OTU_4746 | 0.04% | - | - | - | - | - | - | *Proteobacteria* | *Alphaproteobacteria* | *Rhizobiales* | *Beijerinckiaceae* |  | |
| OTU_4791 | 0.22% | - | - | - | - | - | - | *Proteobacteria* | *Alphaproteobacteria* | *Caulobacterales* | *Caulobacteraceae* | *Phenylobacterium* | |
| OTU_4793 | 0.05% | - | - | - | -0.77 | - | -0.63 | *Proteobacteria* | *Alphaproteobacteria* | *Rhizobiales* | *Beijerinckiaceae* | *Microvirga* | |
| OTU_4844 | 3.89% | -0.57 | - | - | - | - | -0.57 | *Proteobacteria* | *Gammaproteobacteria* | *Pseudomonadales* | *Pseudomonadaceae* | *Pseudomonas* | |

‘-’ represented there was no correlation with the bioactive ingredients. Bold fonts represented positive correlation. RA: Relative abundance. For: Formononetin; CAG: Calycosin-7-β-glucoside; CA: Calycosin; AstI: Astragalosides I; AstII: Astragalosides II; Ast III: Astragalosides III.

**Table S6** Spearman correlation analysis of the shared bacterial OTUs in roots with the contents of bioactive ingredients.

| Bacteria_Root | | Correlation analysis | | | | | | Taxonomy | | | | |
| --- | --- | --- | --- | --- | --- | --- | --- | --- | --- | --- | --- | --- |
| OUT_ID | RA | AstI | AstII | AstIII | CA | CAG | For | Phylum | Class | Order | Family | Genus |
| OTU_8 | 2.59% | -0.68 | -0.56 | - | - | - | - | *Proteobacteria* | *Gammaproteobacteria* | *Pseudomonadales* | *Pseudomonadaceae* | *Pseudomonas* |
| **OTU_10** | 11.78% | - | - | - | **0.60** | - | - | *Proteobacteria* | *Alphaproteobacteria* | *Rhizobiales* | *Rhizobiaceae* | *Phyllobacterium* |
| OTU_11 | 10.22% | - | - | - | -0.58 | -0.59 | -0.57 | *Actinobacteria* | *unidentified_Actinobacteria* | *Micrococcales* | *Microbacteriaceae* | *Microbacterium* |
| **OTU_30** | 0.33% | - | - | - | **0.58** | **-** | - | *Actinobacteria* | *unidentified_Actinobacteria* | *Micrococcales* | *Microbacteriaceae* | *Frigoribacterium* |
| **OTU_34** | 1.88% | - | - | -0.53 | **0.68** | **0.57** | - | *Proteobacteria* | *Alphaproteobacteria* | *unidentified* | *unidentified_Alphaproteobacteria* | *Inquilinus* |
| OTU_45 | 0.77% | -0.68 | - | - | - | - | - | *Actinobacteria* | *unidentified_Actinobacteria* | *Micrococcales* | *Microbacteriaceae* | *Agromyces* |
| OTU_58 | 0.74% | - | - | - | - | - | - | *Firmicutes* | *Bacilli* | *Bacillales* | *Planococcaceae* | *Domibacillus* |
| OTU_65 | 3.04% | - | - | - | - | - | - | *Actinobacteria* | *unidentified_Actinobacteria* | *Micrococcales* | *Micrococcaceae* |  |
| OTU_123 | 0.12% | - | - | - | -0.53 | - | - | *Actinobacteria* | *unidentified_Actinobacteria* | *Micrococcales* | *Microbacteriaceae* | *Microbacterium* |
| OTU_145 | 1.61% | - | - | - | -0.6 | - | -0.76 | *Proteobacteria* | *Alphaproteobacteria* | *Rhizobiales* | *unidentified_Rhizobiales* | *Devosia* |
| OTU_218 | 0.57% | -0.53 | - | - | -0.67 | -0.54 | -0.57 | *Proteobacteria* | *Alphaproteobacteria* | *Rhizobiales* | *Hyphomicrobiaceae* | *Hyphomicrobium* |
| OTU_235 | 0.09% | - | - | - | - | - | - | *Proteobacteria* | *Alphaproteobacteria* | *Rhizobiales* | *Beijerinckiaceae* | *Methylobacterium* |
| OTU_239 | 0.74% | - | - | - |  | - | -0.61 | *Proteobacteria* | *Alphaproteobacteria* | *Sphingomonadales* | *Sphingomonadaceae* | *Novosphingobium* |
| OTU_250 | 0.93% | - | - | - | -0.52 | - | -0.72 | *Proteobacteria* | *Alphaproteobacteria* | *Rhizobiales* | *unidentified_Rhizobiales* | *Devosia* |
| OTU_262 | 0.46% | -0.57 | -0.59 | - | - | - | -0.77 | *Firmicutes* | *Bacilli* | *Bacillales* | *Planococcaceae* | *Planomicrobium* |

‘-’ represented there was no correlation with the bioactive ingredients. Bold fonts represented positive correlation. RA: Relative abundance. For: Formononetin; CAG: Calycosin-7-β-glucoside; CA: Calycosin; AstI: Astragalosides I; AstII: Astragalosides II; Ast III : Astragalosides III.

**Table S7** Spearman correlation analysis of the shared fungal OTUs in rhizosphere and roots with the contents of bioactive ingredients.

| Fungi_Rhizopshere | | Correlation analysis | | | | | | Taxonomy | | | | |
| --- | --- | --- | --- | --- | --- | --- | --- | --- | --- | --- | --- | --- |
| OUT_ID | RA | AstI | AstII | AstIII | CA | CAG | For | Phylum | Class | Order | Family | Genus |
| **OTU_16** | 1.10% | - | - | - | -0.53 | - | - | *Basidiomycota* | *Tremellomycetes* | *Filobasidiales* | *Filobasidiaceae* | *Naganishia* |
| OTU_173 | 0.06% | - | - | - | - | - | - | *Basidiomycota* | *Cystobasidiomycetes* | *Cystobasidiales* | *Cystobasidiaceae* | *Cystobasidium* |
| OTU_1464 | 0.01% | - | - | - | - | - | - | *Ascomycota* | *Eurotiomycetes* | *Eurotiales* | *Aspergillaceae* | *Aspergillus* |
| **OTU_1668** | 0.01% | - | - | - | -0.52 | - | - | *Unidentified_fungi* | *Unidentified_fungi* | *Unidentified_fungi* | *Unidentified_fungi* | *Unidentified_fungi* |
| Fungi_Root | | Correlation analysis | | | | | | Taxonomy | | | | |
| OUT_ID | RA | AstI | AstII | AstIII | CA | CAG | For | Phylum | Class | Order | Family | Genus |
| **OTU_840** | 0.01% | - | -0.53 | - | - | - | - | *Ascomycota* | *Dothideomycetes* | *Capnodiales* | *Mycosphaerellaceae* | *Polythrincium* |
| OTU_931 | 0.22% | - | - | - | - | - | - | *Unidentified_fungi* | *Unidentified_fungi* | *Unidentified_fungi* | *Unidentified_fungi* | *Unidentified_fungi* |
| OTU_1298 | 3.34% | - | - | - | - | - | - | *Unidentified_fungi* | *Unidentified_fungi* | *Unidentified_fungi* | *Unidentified_fungi* | *Unidentified_fungi* |
| OTU_1976 | 19.04% | - | - | - | - | - | - | *Unidentified_fungi* | *Unidentified_fungi* | *Unidentified_fungi* | *Unidentified_fungi* | *Unidentified_fungi* |
| **OTU_2427** | 0.01% | - | - | - | -0.53 | - | - | *Ascomycota* | *Dothideomycetes* | *Capnodiales* | *Mycosphaerellaceae* | *Polythrincium* |
| OTU_2456 | 0.02% | - | - | - | - | - | - | *Unidentified_fungi* | *Unidentified_fungi* | *Unidentified_fungi* | *Unidentified_fungi* | *Unidentified_fungi* |
| OTU_2644 | 0.03% | - | - | - | - | - | - | *Unidentified_fungi* | *Unidentified_fungi* | *Unidentified_fungi* | *Unidentified_fungi* | *Unidentified_fungi* |
| OTU_3572 | 0.45% | - | - | - | - | - | - | *Unidentified_fungi* | *Unidentified_fungi* | *Unidentified_fungi* | *Unidentified_fungi* | *Unidentified_fungi* |
| OTU_3619 | 0.01% | - | - | - | - | - | - | *Unidentified_fungi* | *Unidentified_fungi* | *Unidentified_fungi* | *Unidentified_fungi* | *Unidentified_fungi* |

‘-’ represented there was no correlation with the bioactive ingredients. Bold fonts represented positive correlation. RA: Relative abundance. For: Formononetin; CAG: Calycosin-7-β-glucoside; CA: Calycosin; AstI: Astragalosides I; AstII: Astragalosides II; Ast III : Astragalosides III.


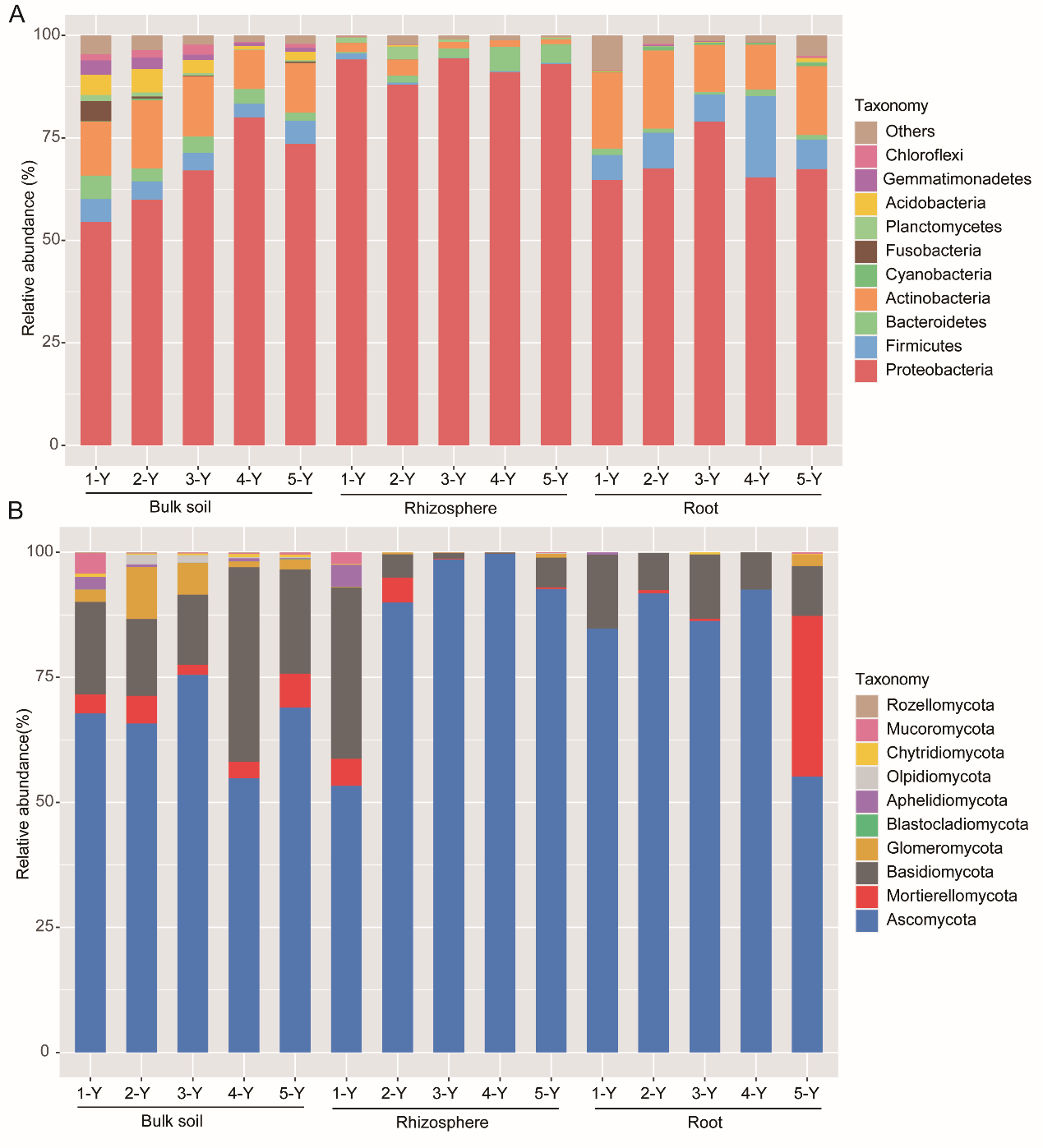


**Figure S1** The relative abundance of top 10 phyla of bacterial **(A)** and fungal **(B)** community composition of different sampling compartments across different cultivation years.


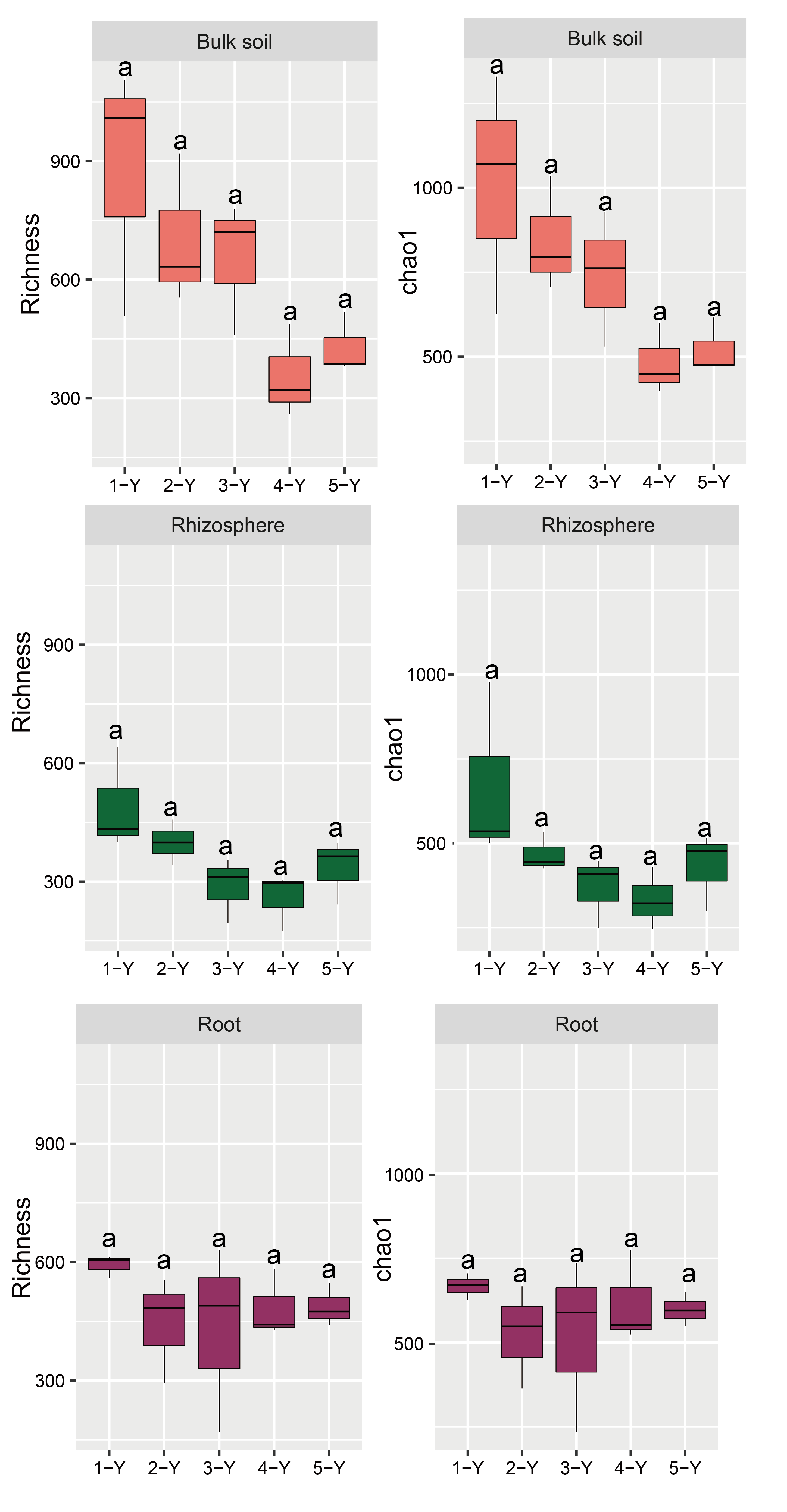


**Figure S2** The alpha-diversity (Richness and Chao1 index) of bacteria microbiomes across different cultivation years. The analysis of variance (ANOVA) was used to compare the Richness and Chao1 index in different cultivation years (*P<0.05*).


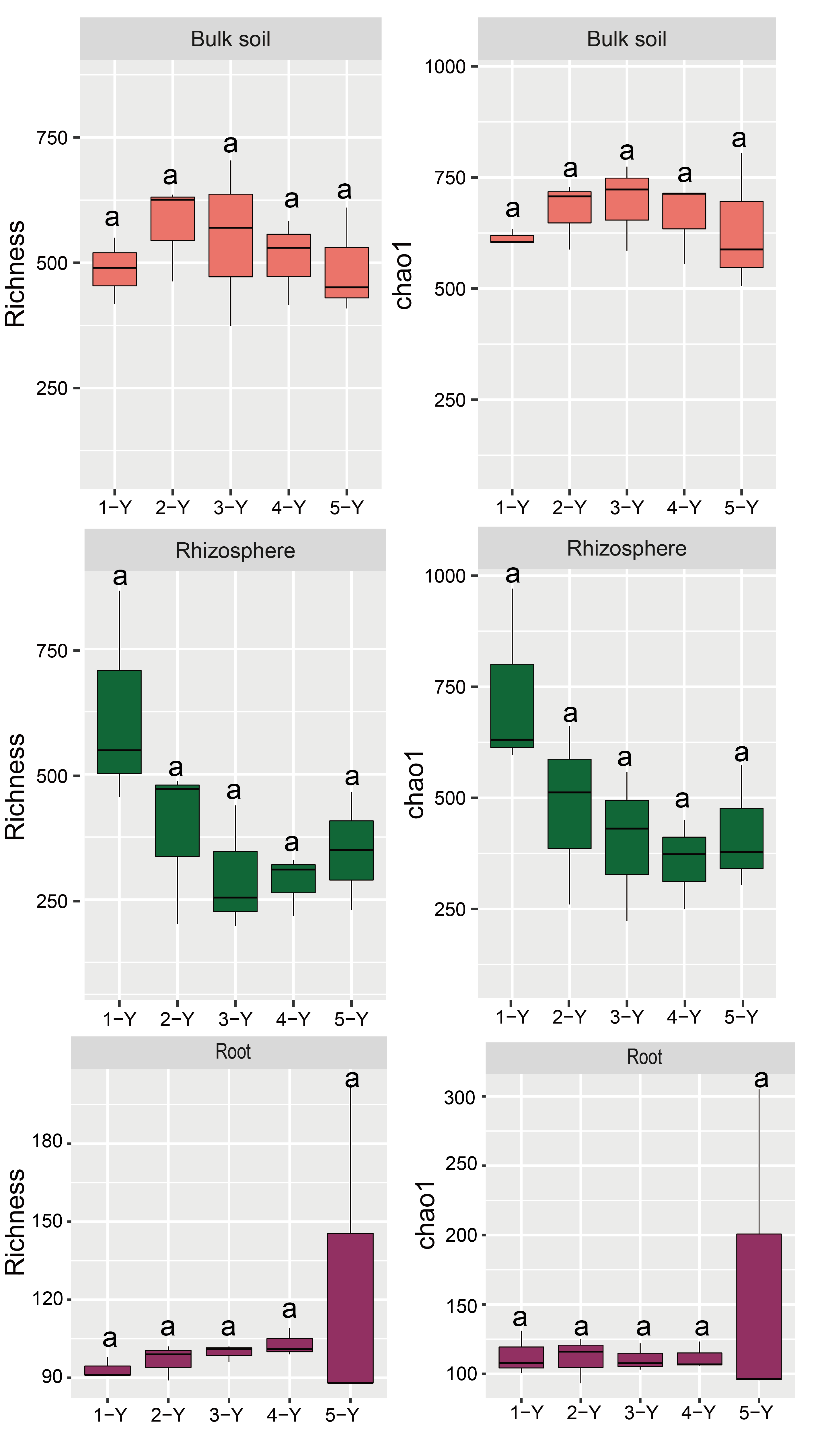


**Figure S3** The alpha-diversity (Chao1 index and Richness) of fungi microbiomes across different cultivation years. The analysis of variance (ANOVA) was used to compare the Richness and Chao1 index in different cultivation years (*P<0.05*).


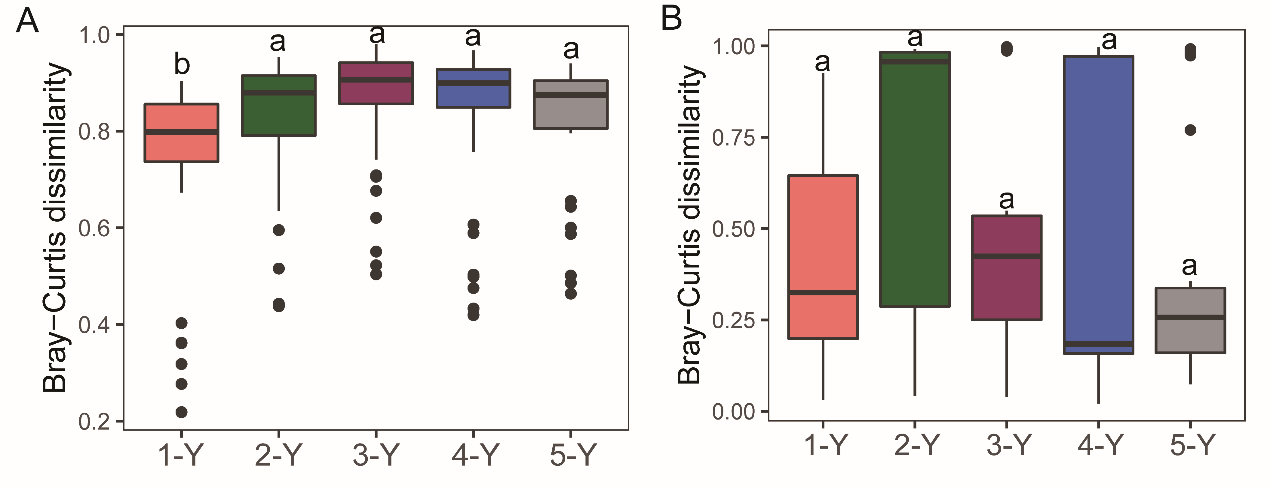


**Figure S4** Boxplots of root-associated bacterial **(A)** and fungal **(B)** community similarity among different cultivation years. Different letters indicate significant differences (*P < 0.05*, Kruskal-wallis test).

**
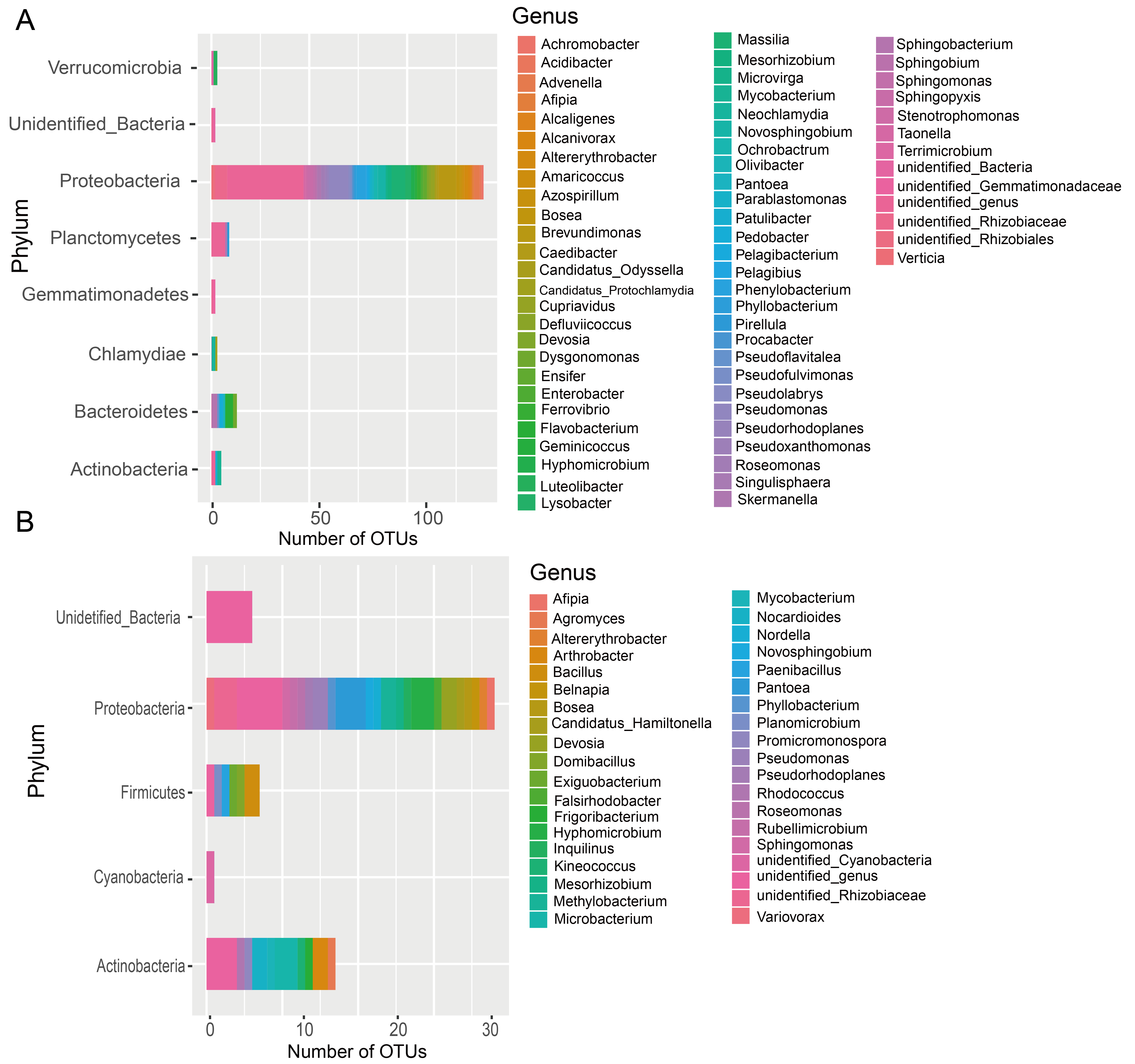
**

**Figure S5** The differentially bacterial OTUs in the rhizosphere **(A)** and root endosphere **(B)** at the genus level.


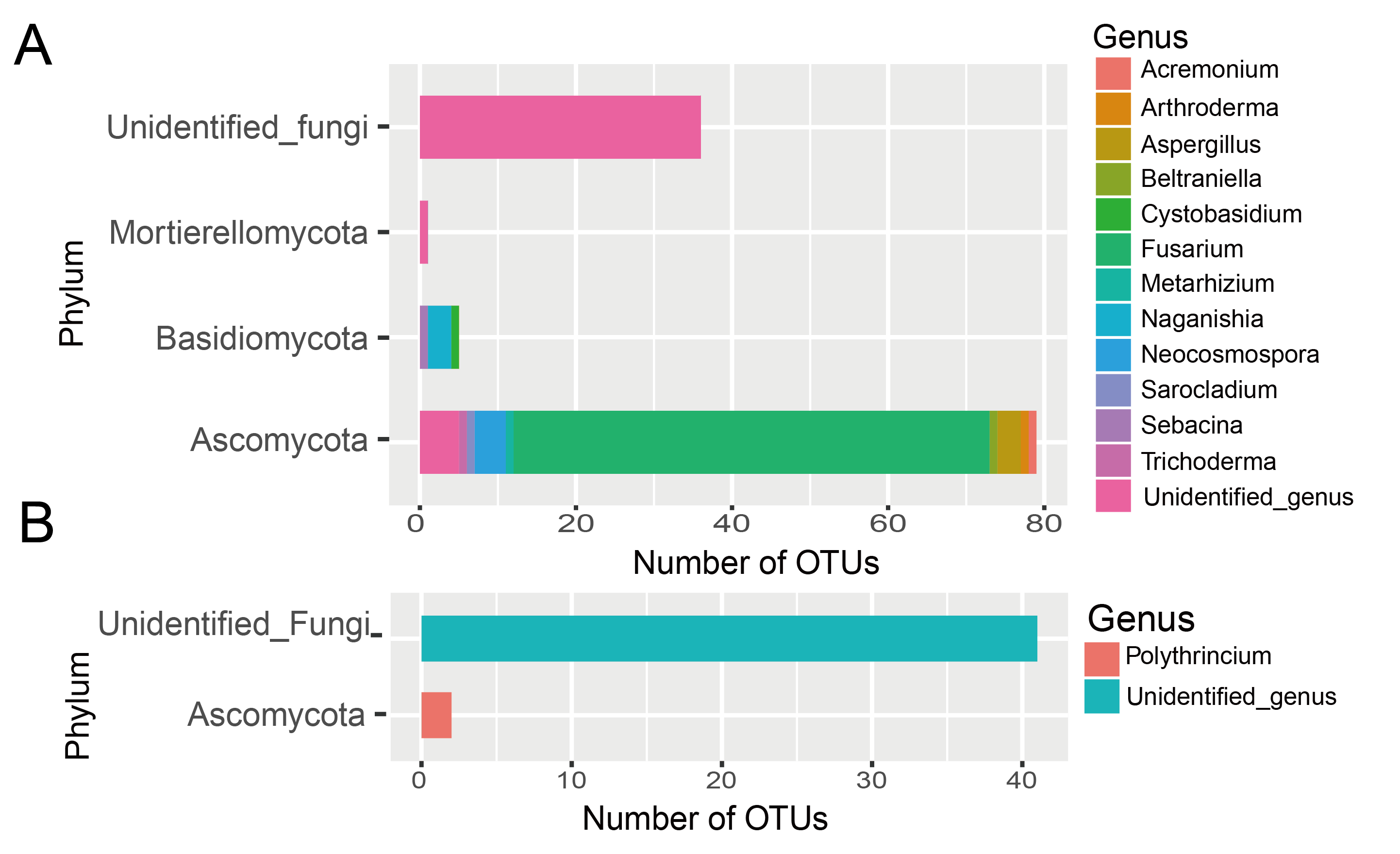


**Figure S6** The differentially fungal OTUs in the rhizosphere **(A)** and root endosphere **(B)** at the genus level.


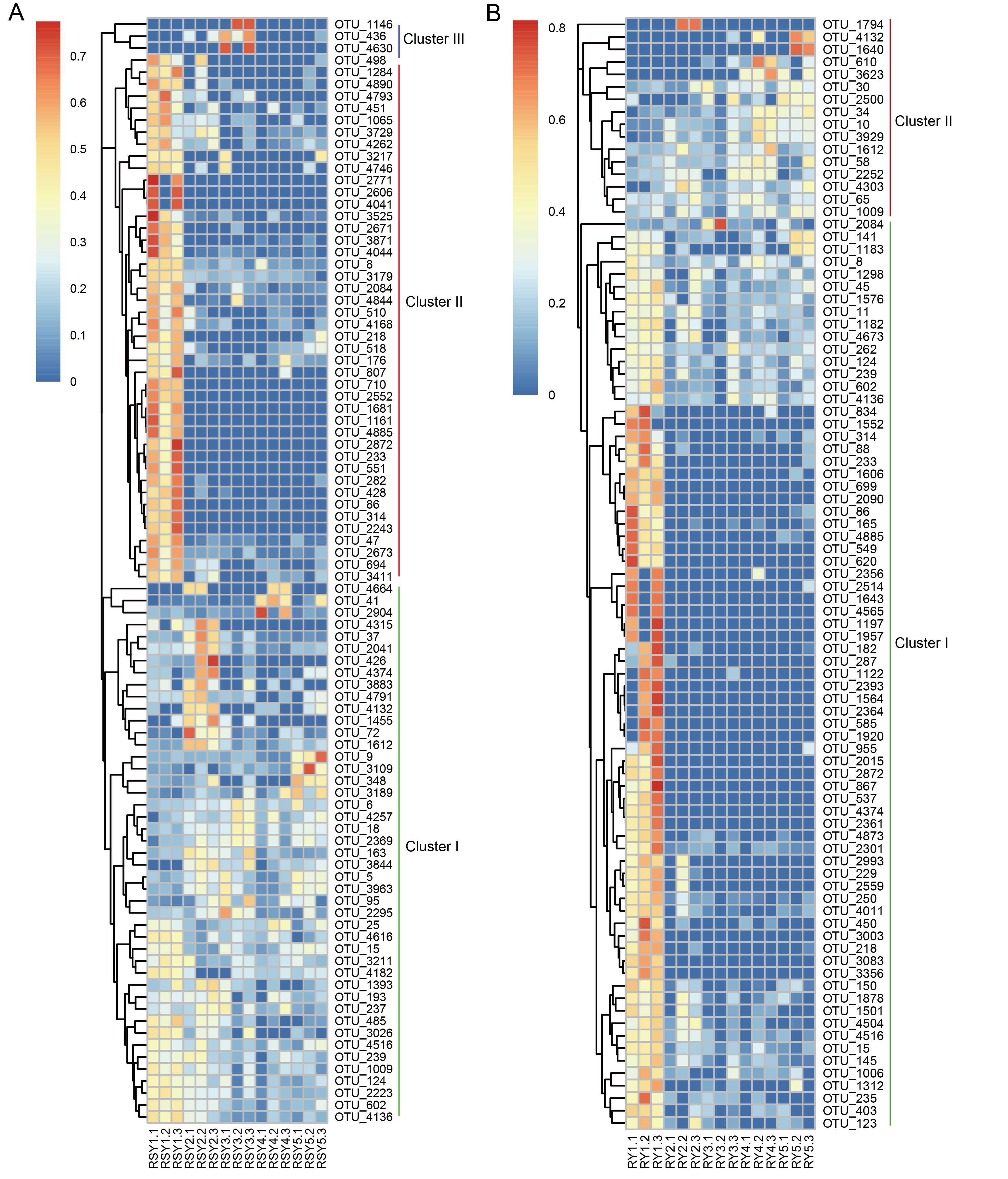


**Figure S7** Heatmap of the relative abundance of bacterial OTUs with significant variation among different cultivation year in rhizosphere **(A)** and root endosphere **(B)**.


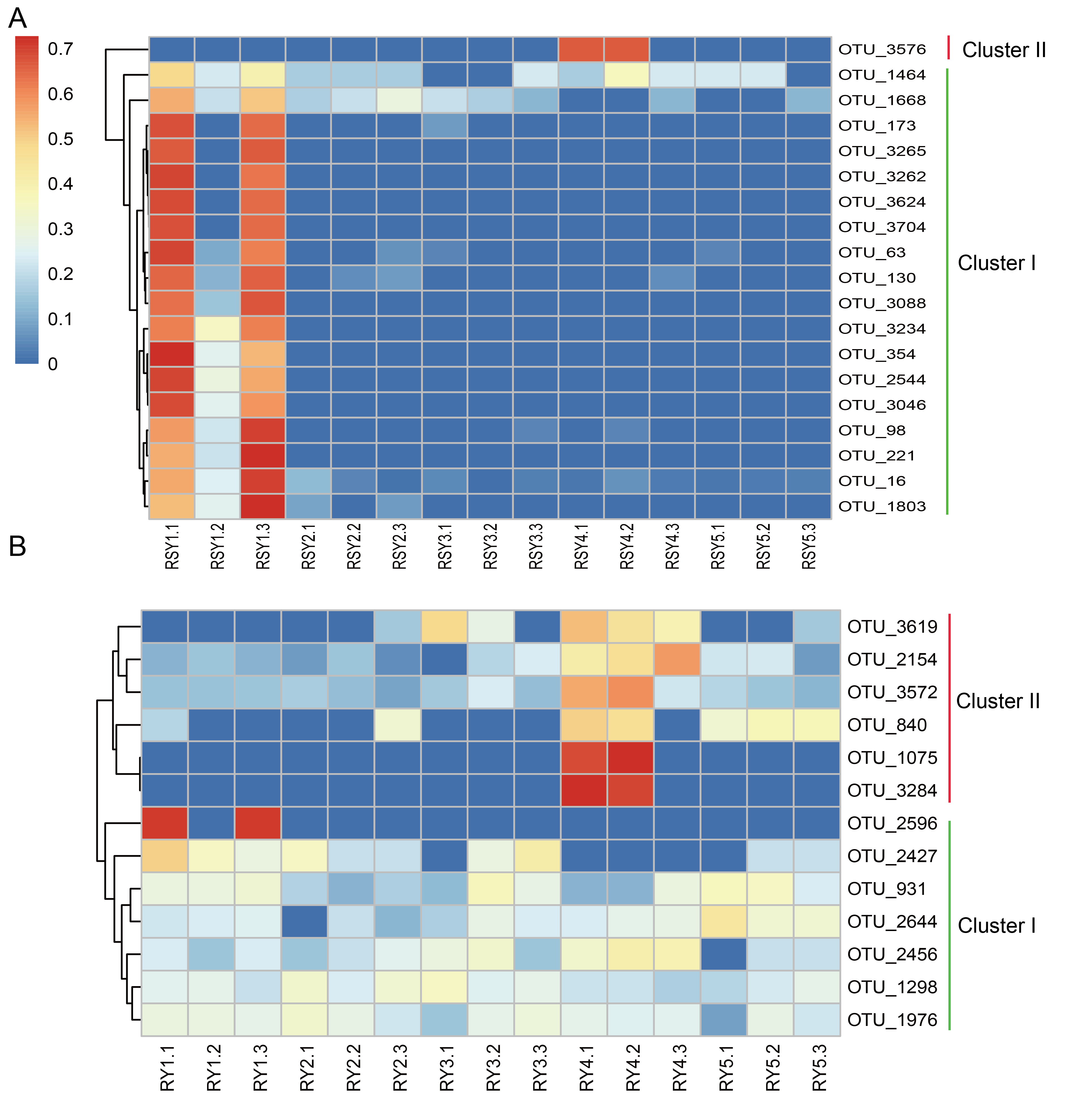


**Figure S8** Heatmap of the relative abundance of fungal OTUs with significant variation among different cultivation year in rhizosphere **(A)** and root endosphere **(B)**.


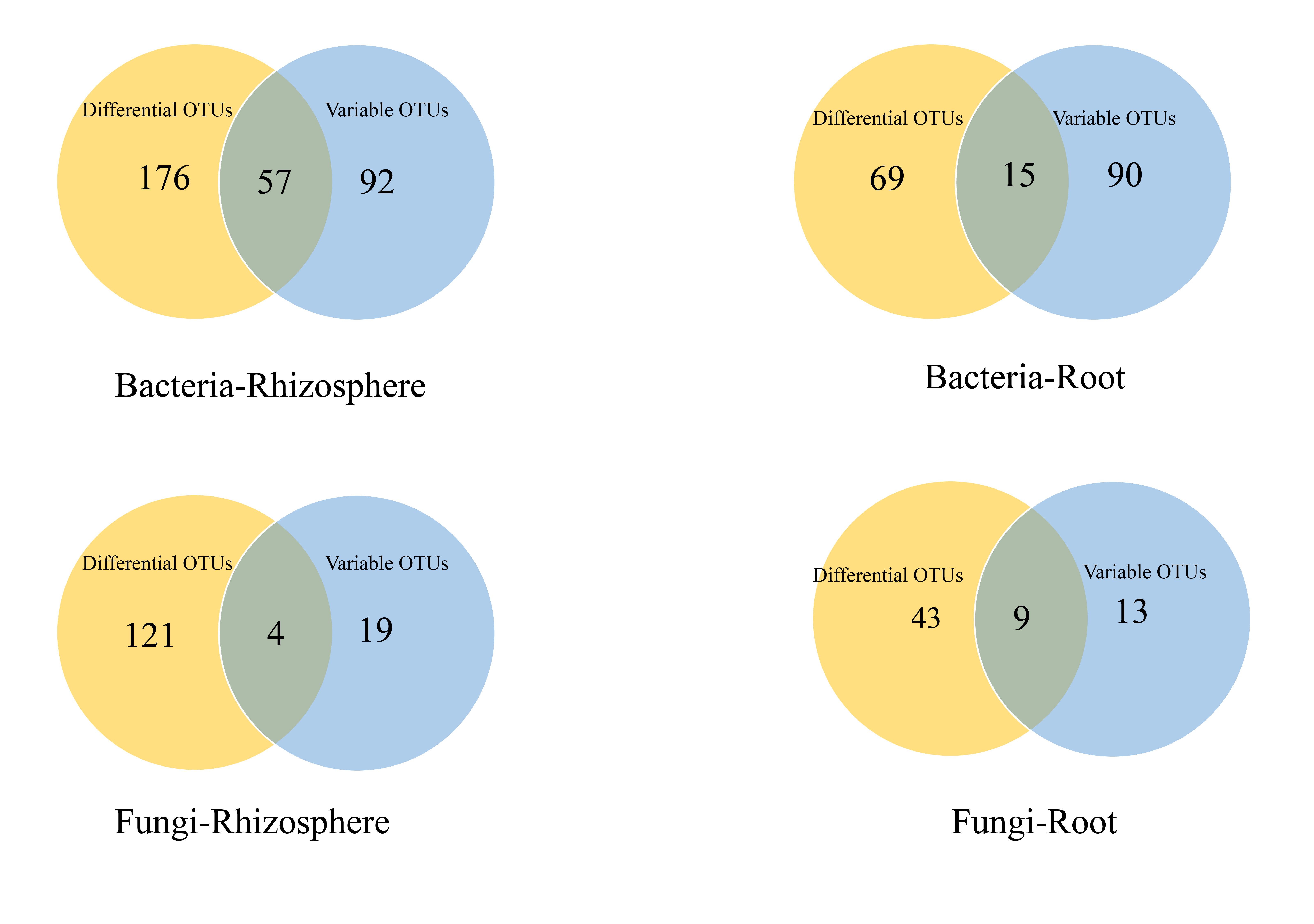


**Figure S9** Venn diagrams of the shared OTUs in root-associated bacteria and fungi. Differential OTUs represented the OTUs significantly enriched in rhizosphere and root; Variable OTUs represented the rhizospheric and root endosphere OTUs significantly varied across different cultivation years.
